# Exploring the Interplay Between Message Format, Need for Cognition and Personal Relevance on Processing Messages About Physical Activity: a Two-Arm Randomized Experimental Trial

**DOI:** 10.1007/s12529-022-10107-4

**Published:** 2022-06-10

**Authors:** Camille E. Short, Rik Crutzen, Emma M. Stewart, Jessica O’Rielly, Mathew Dry, Andrew Skuse, Pascale Quester, Amanda L. Rebar, Corneel Vandelanotte, Mitch J. Duncan, Andrew Vincent

**Affiliations:** 1 Freemasons Foundation Centre for Men’s Health, School of Medicine, University of Adelaide, Adelaide, Australia; 2 Melbourne School of Psychological Sciences and Melbourne School of Health Sciences, University of Melbourne, Melbourne, VIC Australia; 3 Department of Health Promotion/CAPHRI, Maastricht University, Maastricht, The Netherlands; 4 Department of Experimental Psychology, Justus Liebig University Giessen, Giessen, Germany; 5 School of Psychology, University of Adelaide, Adelaide, Australia; 6 Anthropology and Development Studies, University of Adelaide, Adelaide, Australia; 7 Adelaide Business School, The University of Adelaide, Adelaide, Australia; 8 Physical Activity Research Group, Appleton Institute, School of Health, Medical and Applied Sciences, Central Queensland University, Rockhampton, Australia; 9 School of Medicine & Public Health, Faculty of Health and Medicine, The University of Newcastle, University Drive, Callaghan, NSW Australia; 10 Priority Research Centre for Physical Activity and Nutrition, The University of Newcastle, University Drive, Callaghan, NSW Australia

**Keywords:** Physical activity, Health communication, Individuality, Persuasive communication

## Abstract

**Background:**

According to the Elaboration Likelihood Model, persuasion can occur via two different routes (the central route and peripheral route), with the route utilized dependent on factors associated with motivation and ability. This study aimed to explore the moderating role of need for cognition (NFC) and perceived relevance on the processing of physical activity messages designed to persuade via either the central route or the peripheral route.

**Method:**

Participants (*N* = 50) were randomized to receive messages optimized for central route processing or messages optimized for peripheral route processing. Eye-tracking devices were used to assess attention, which was the primary outcome. Message perceptions and the extent of persuasion (changes in physical activity determinants) were also assessed via self-report as secondary outcomes. Moderator effects were examined using interaction terms within mixed effects models and linear regression models.

**Results:**

There were no detected interactions between condition and NFC for any of the study outcomes (all *p*s > .05). Main effects of personal relevance were observed for some self-report outcomes, with increased relevance associated with better processing outcomes. An interaction between need for cognition and personal relevance was observed for perceived behavioral control (*p* = 0.002); greater relevance was associated with greater perceived behavioral control for those with a higher need for cognition.

**Conclusion:**

Matching physical activity messages based on NFC may not increase intervention efficacy. Relevance of materials is associated with greater change in physical activity determinants and may be more so among those with a higher NFC.

**Supplementary Information:**

The online version contains supplementary material available at 10.1007/s12529-022-10107-4.

## Introduction

Physical inactivity is a widespread global problem associated with increased morbidity, premature mortality, and substantial economic burden [1]. To address this at scale, cost-effective and wide-reaching physical activity interventions are recommended [2]. Digital and print media interventions (e.g., websites, mobile applications, pamphlets, and booklets) have the potential to reach large numbers of individuals at a relatively low cost [3, 4]. Although originally delivered as mass-marketed one-size-fits-all interventions, they are increasingly personalized to reflect either the subgroups individuals belong to (i.e., a targeted intervention) or the characteristics of each individual specifically (i.e., a tailored intervention). Several systematic reviews and meta-analyses have shown that customizing print and digital interventions in these ways lead to larger intervention effects on health-related behaviors [5–7].

Personal relevance is thought to be the primary factor leading to the increased efficacy of tailored health messages [8]. This is based on the Elaboration Likelihood Model of Persuasion [9], which suggests that there are two routes to persuade someone (i.e., influencing beliefs and/or intentions in desired direction), the central route and the peripheral route. The central route is used when participants have the ability and motivation to process information elaboratively. This type of in-depth processing is thought to result in enduring changes to attitudes and be most likely to occur when messages are perceived as personally relevant. The peripheral route, on the other hand, reflects the use of simpler processes, such as the use of heuristics, biases, and affect, and requires fewer resources and less motivation to process information. Persuasion can also occur via this route, though it is believed by some to be less resistant to counter persuasion [10]. According to popular social cognitive models of behavior change, intentions are formed based on deliberation of beliefs (e.g., attitudes, self-efficacy beliefs), and behavior change occurs through the translation of these intentions into actions [11]. On this basis, interventions that successfully lead to the development of enduring attitudes should then lead to consistent intentions and in turn a higher likelihood of sustained changes in behavior [12]. As such, increasing the likelihood of central route persuasion, in particular by ensuring personal relevance, has been a primary goal of health promotion campaigns [13]. However, there are factors other than personal relevance that may influence motivation and ability to process information (e.g., educational attainment, level of focus or distraction). Furthermore, evidence from other areas (e.g., advertising) demonstrates that messages designed for peripheral route processing can exert powerful influences on behavior and its antecedents, especially with repeat or timely exposure [14, 15]. It may therefore be possible to increase the efficacy of physical activity promotional campaigns by seeking to persuade via the peripheral route, and/or by identifying other factors that may impact on ability and motivation to process information via the central route.

One potentially important factor is “need for cognition.” Need for cognition (NFC thereafter) is a personality characteristic reflecting a person’s tendency to engage in and enjoy effortful cognitive activities [16, 17]. Individuals with a high NFC enjoy cognitive tasks and show greater motivation to engage in the elaborate processing of information [16–19]. In contrast, individuals with lower NFC have a tendency to prefer processing information less systematically, and as such are more likely to rely on simple cues and heuristics, like the endorsement of others (e.g., celebrities or experts) or social comparison processes (“they are enjoying it, I might enjoy it too”) [16–19]. Hence, it is possible that high NFC individuals will be most likely persuaded by messages optimized for central route processing, and low NFC individuals persuaded by messages designed for peripheral route processing (i.e., when messages are “matched” to their NFC). A recent review found some evidence in support for this [20], though there was only one physical activity study [21] and findings were mixed. Favorable outcomes were achieved among those with lower NFC when messages were matched to processing style (supporting matching theory); however, message type was not associated with persuasion outcomes among those with a higher NFC (i.e., no difference when matched or unmatched) [21].

One limitation of research to date has been that the majority of studies have treated NFC as a binary construct [20]. This is not ideal, as NFC is normally distributed and exists along a continuum [22]. Another issue is that studies have not considered the impact of perceived personal relevance. This would seem prudent, given that both NFC and perceived message relevance are considered key motivators of message processing, and that it is hypothesized that they likely influence each other. According to Cacioppo [17, 23], processing differences based on NFC are likely to be most evident when health promotion materials are perceived as moderately relevant. When messages are either very low or very high in relevance, individuals are likely to process information in a similar way, regardless of NFC. This is because relevance has a strong influence on motivation to process information elaborately. When relevance is very low, so too is motivation to process information in an in-depth way. Likewise, when relevance is very high, individuals have increased motivation to process information elaborately. Whereas when relevance is moderate, the influence of relevance on motivation is more subdued and need for cognition is more likely to exert and influence on message processing.

The primary aim of the current study was to examine the moderating role of NFC on the processing of physical activity materials optimized for either central or peripheral route processing. For the purpose of this study, message processing was broadly defined as the selection of stimuli to attend to, the interpretation of stimuli, and whether or not persuasion occurred [24]. Given that physical activity promotion materials should be theory-informed and designed to target key modifiable determinants of physical activity behavior [25], we define persuasion in this context as changes to theory-based determinants of physical activity at the individual level (e.g., changes in attitudes). We hypothesized that individuals who received messages more aligned with their need for cognition would show greater attention towards the messages, report more positive perceptions, and display higher levels of persuasion than individuals who received unaligned messages. Our secondary aim, which was exploratory, was to examine the role of personal relevance as an influence on the relationship between NFC and message processing. We expected NFC and personal relevance to interact when influencing message processing, such that NFC would be most influential when perceived personal relevance was moderate. At very low or very high levels of relevance, NFC was not anticipated to be associated with message processing.

## Method

### Study Design

This trial was a two-group randomized experimental study. Participants were randomized 1:1 to receive either stimuli optimized for central route processing or stimuli optimized for peripheral route processing. Outcomes were assessed during the presentation of stimuli on a computer monitor (using eye tracking devices) and pre- and post-exposure using self-administered questionnaires. Total gaze duration within pre-specified areas of interest (AOI), which is a valid and reliable measure of visual attention [26] was used as the primary outcome measure. Ethics approval was obtained from the School of Psychology Human Research Ethics Sub Committee at the University of Adelaide. To facilitate accurate replication, study materials, including intervention stimuli, can be downloaded from the study’s repository at the Open Science Framework [https://osf.io/cj4ze/]. Reporting of the study is in line with the CONSORT statement for randomized trials [27]

### Setting and Participants

The study was conducted at the University of Adelaide between March and April 2016. Participants included adults aged 18 to 60, who could read and write in English, and attend the testing site during business hours (9am–5 pm Monday-Friday). Inclusion in the study required that they could clearly view a computer screen without the assistance of glasses (contact lenses permitted), had not had laser eye surgery, and were not already participating in 30 min of moderate-vigorous physical activity on five or more days per week (assessed via self-report). Participants were recruited using a variety of methods, including posts on Facebook and Gumtree (i.e., a classified advertisement site), and the use of hard copy flyers posted around the University. Participants received a $50 visa debit card as reimbursement.

### Accrual and Randomization

All recruitment material contained a link to the study website where potential participants could complete the screening questionnaire and register for the study if eligible. Recruitment was paused within 5 days of commencement due to the large number of eligible responders.

Given that the majority of candidates were assumed to be university students and may therefore be more likely to exhibit higher need for cognition [28], an accrual protocol was devised a priori to maximize the variance of need for cognition scores in enrolled individuals. In brief, a 3-item measure of need for cognition [20] was included in the screening questionnaire. The first five candidates were accrued, and subsequently the Kullback–Leibler (KL) divergence between the distribution of the 3-item sum and the uniform distribution was calculated. Thereafter, candidates were accrued automatically if the KL divergence improved; otherwise, the chance of accrual was set at 25%, thereby increasing the variance in NFC scores. Upon accrual, individuals were randomly distributed between central versus peripheral route intervention groups using a randomized block design, stratified by the 3-item sum dichotomized (low vs high) NFC score, with random block lengths of 2, 4, or 6. The randomization protocol was designed by author AV and actioned by author CES.

The first 50 candidates accrued were contacted by the research team to make an appointment time. If an appointment time could not be made (due to no response or unavailability) or if the participant failed to show up, the next accrued candidate on the list was contacted and asked to schedule an appointment. This continued until 50 individuals had been tested.

### Experimental Conditions

Both sets of experimental materials were developed to target the same determinants of physical activity behavior (intention, attitudes, and perceived behavioral control as outlined by the Theory of Planned Behavior [29]) and convey the same key take-home messages (see Supplementary Material 1). Theory of Planned behavior was chosen as the guiding theoretical framework as it is one of the most commonly utilized theories in physical activity promotion using print and digital media [4, 30], and its use is associated with increased effect sizes [30]. How the key messages were communicated differed in each experimental condition, as described below. Quality of materials was assured through rigorous pre-testing (*n* = 21) prior to the trial (see https://osf.io/cj4ze/).

#### Central Route Materials

**Fig. 1:**
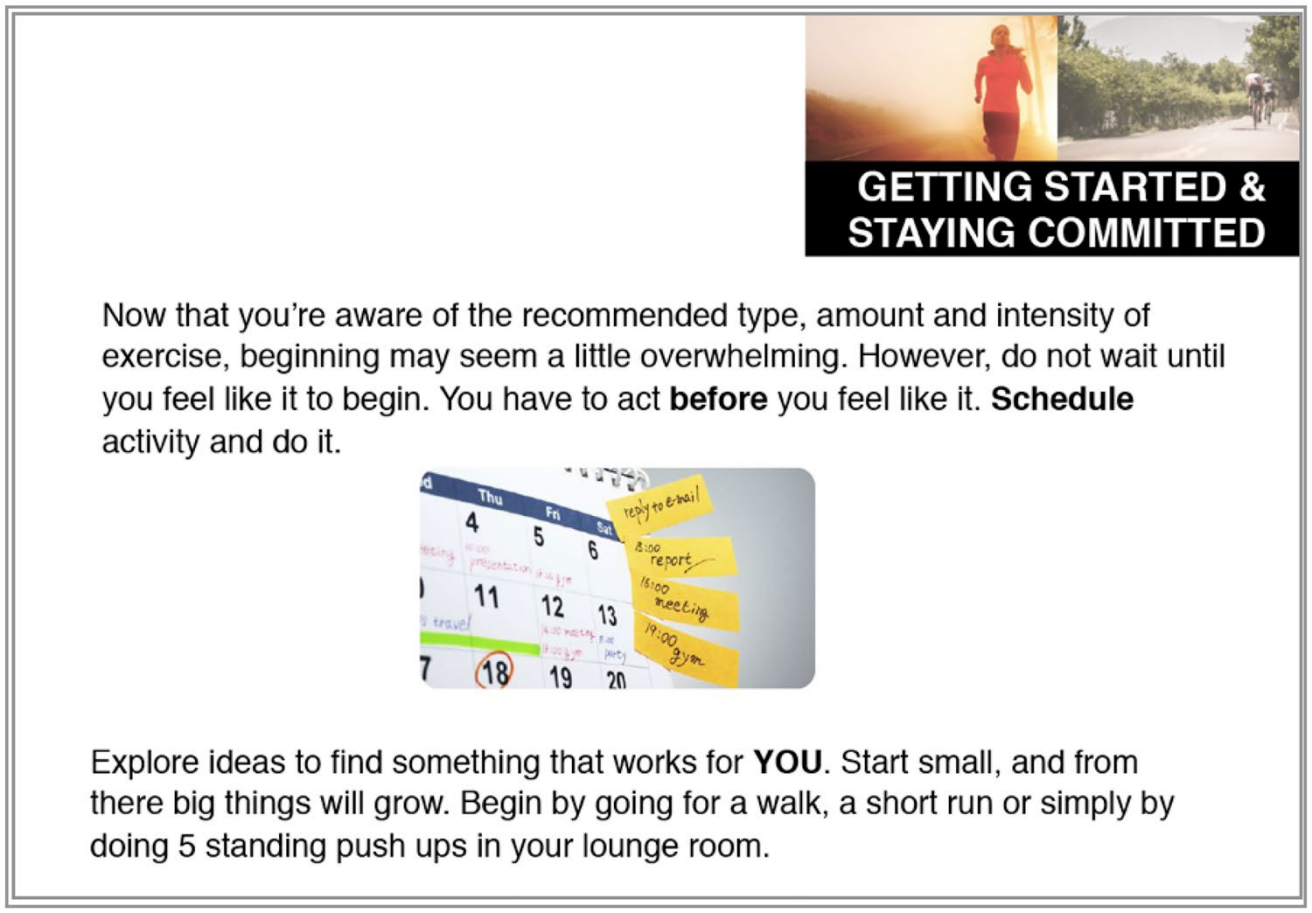
Example slide from the central route experimental condition. This slide was designed to influence experiential attitudes and perceived behavioral control by encouraging message recipients to set graded tasks that suit them

Materials in this condition emphasized facts relating to physical activity benefits and behavior change and presented arguments in a direct manner. The information was presented in a modular format, simulating common educational materials. Compared to the other condition, more in-depth information was provided, including information on mechanisms of action (e.g., explaining *how* exercise leads to less stress). Images and color were used minimally and were only a key focal point when they were instructive (e.g., graph showing the relationship between health benefits and exercise intensity) or neutral (e.g., reinforce text without evoking emotion). The materials consisted of 11 slides in total. See Fig. 1 for an example slide.

#### Peripheral Route Materials

**Fig. 2:**
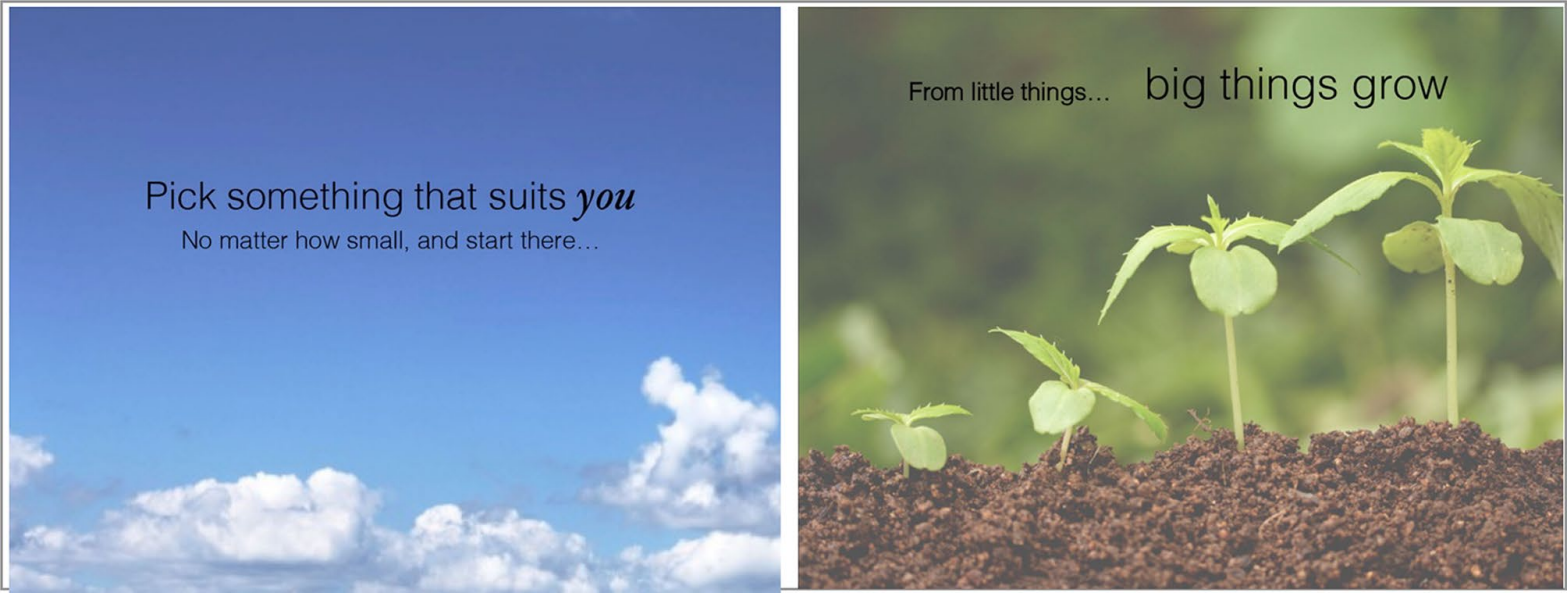
Example slides from the peripheral route experimental condition. These slides were also designed to influence experiential attitudes and perceived behavioral control by encouraging message recipients to set graded tasks that suit them

The materials in this condition were presented in a more simplistic manner and were designed to evoke positive feelings and engage the reader via a familiar narrative. Compared to the central route material, images and color were relied on heavily, with minimal text. The materials consisted of 14 slides in total. See Fig. 2 for an example of slides.

## Measures

### Participant Characteristics

Assessed demographic factors included gender, age, employment status, highest level of education, post-tax household income level, and relationship status. Body mass index (BMI; kg/m^2^) was calculated based on self-reported height and weight. Physical activity was assessed using an adapted version of the Godin Leisure-Time Exercise Questionnaire [31, 32], with vigorous activity minutes weighted by two to account for additional benefits. Physical activity habit strength was assessed with the Behavioral Automaticity Index [33]. Previous exposure to physical activity messages was assessed using a purpose-built single item “How much information or advice about building healthy physical activity habits would you say you have read or viewed in the past?” with six response options ranging from none to a substantial amount.

### Outcomes

#### Attention

When viewing intervention material on the monitor, participants’ eye movements were recorded as an indicator of attention. Total gaze duration within pre-specified areas of interest (AOI; i.e., all graphics and text) was used as the primary measure. The proportion of gaze time within an AOI, relative to total slide viewing time (AOI ratio), was also assessed. Gaze duration within AOIs is considered a valid and reliable measure of visual attention [26] and was chosen as the primary outcome as attention is considered a core component of message processing and may be a prerequisite for influencing user perceptions, attitudes, and intentions [34].

Eye movements were recorded with an Arrington ViewPoint EyeTracker system (Arrington Research), sampling at 220 Hz with a spatial precision of 0.25° of visual angle. The location of gaze was sampled every 4.4 ms. Gaze duration was calculated as the number of samples recorded within that AOI, multiplied by the sampling rate. This allowed for a calculation of raw time (in ms) of gaze in each AOI and the total time spent viewing each slide.

In addition, to help visualize how participants attended to the materials, heatmaps were calculated from gaze points, represented as *x*,*y* scatter density. Data were plotted for participants within the top tertile (highest in NFC) versus the bottom tertile (lowest in NFC).

#### Physical Activity Determinants

Intentions, attitudes, and perceived behavioral control were assessed pre- and post-exposure using standard self-report items, in line with suggestions by Ajzen [35, 36]. Scale scores were created for each determinant by summing items together, with higher scores equating to more positive responses. An overview of the measures used, including items and response scales, is provided in Supplementary Material 2.

#### User Experience and Perceptions

All user experience and perception measures were assessed during the post-exposure questionnaire.

##### Quality of Experience

An adapted version of the enjoyment of website experiences scale [37]was used to assess participants’ experience of viewing the physical activity materials. The measure was adapted to say “while viewing the materials” rather than “while visiting the website.” The measure is comprised of twelve items, four measuring the subscales of engagement, four measuring positive affect, and four items measuring fulfillment. Item scores are summed together to form subscale scores (range = 0–24) and an overall user experience score (range = 0–72). Higher scores indicate a more positive user experience. Previous research has demonstrated that this measure has a high degree of reliability and validity [37].

##### Perceived Message Effectiveness

A 3-item perceived effectiveness scale, adapted from Jensen et al. [38], was used to assess the perceived persuasiveness of the message. The three items are “Was the material convincing?”, “Would people your age who are not already active be more likely to become active after reading the information presented?”, and “Would the materials be helpful for convincing your friends and family to become more physically active?” with 4-point response options: definitely no, no, yes, and definitely yes (score range per item; 0–3). Higher sum scores of the three items indicate greater perceived effectiveness (range = 0–9).

##### Perceived Message Informativeness

Participants’ thoughts about the amount of information in the message materials were measured using an adapted version of the perceived message informativeness scale [39]. The scale includes 2 items which require participants to rate their responses on a 5-point scale ranging from strongly disagree to strongly agree. The two items are “The material was informative” and “I learned something from the material presented.” Higher scores indicate greater perceived informativeness (range = 0–8).

### Moderators

#### Need for Cognition

Need for cognition was assessed at baseline using the short-form need for cognition scale [40]. The scale consists of 18 items requiring participants to rate on a 7-point Likert scale, ranging from “strongly disagree” (0) to “strongly agree” (6), the degree to which they believe the statement to be characteristic of them. Responses were summed to create a total NFC score (range = 0–108) with higher scores indicating higher NFC.

#### Perceived Personal Relevance

Perceived personal relevance of the message was assessed post-test using three purpose-built items, with an acceptable Cronbach’s alpha (0.79). Items were scored on a 7-point Likert scale that ranged from “strongly disagree” to “strongly agree.” The items were as follows: “The information provided was very relevant to me”; “The information provided was applicable to my situation”; “The information provided seems like it was written with someone like me in mind.” Personal relevance scores were calculated by summing the responses to the three items together (range = 0–21), with higher scores indicating the participants held higher personal relevance perceptions of the message.

### Sample Size Calculation

We assumed that (i) the difference in prevalence of NFC categories (high vs. low) would not exceed 40% (i.e., ranging from 3:7 to 7:3); (ii) under an appropriate transformation, gaze duration outcome mean would be normally distributed per combination (treatment group × low vs. high NFC) with constant within-group standard deviation (SD); and (iii) the alternative hypothesis of interest consists of high (low) NFC individuals having a gaze duration of 2.5 SD (1.5 SD) when viewing matched materials and 0.5 SD (0.5 SD) spans when viewing mismatched materials. Subsequent simulations indicated that 50 individuals (25 allocated to each experimental condition) would be sufficient to provide at least 80% power to detect an interaction between need for cognition (high vs low) and treatment groups (central vs peripheral) in a linear regression (2-sided alpha = 0.05).

### Statistical Method

Means (standard deviations) and frequencies (percentages) are reported for continuous and discrete participant demographics, respectively, unless otherwise specified. All analyses included only those that completed data collection (*n* = 50).

The analysis of both the primary endpoint, gaze duration within AOIs, and the ratio of gaze duration time within AOIs to total duration consisted of a mixed effects binomial regression model with quadratic parameterization. As gaze duration differed greatly by slide, in both analyses, non-nested random intercepts were included for both individual and slide. Fixed effects consisted of NFC, treatment allocation (central vs peripheral), age, gender (female vs male), moderate-to-vigorous physical activity, prior message exposure, and personal relevance. The interaction between NFC and treatment allocation was of primary interest, and an additional post hoc interaction between NFC and personal relevance was also explored. In these analyses, continuous fixed effects were centered and standardized by the sample mean and standard deviation. For the primary analysis, individuals were analyzed into the group to which they were allocated. There was one individual in the peripheral route condition with a total AOI duration of 229 s, almost three times the next slowest individual in that group (82 s). We believe that this was probably due to English being a second language. There was also one individual who did not receive the correct stimulus. A sensitivity analysis was explored to examine the impact of these protocol deviations, which excluded the individual with English language difficulties and ensured that individuals were analyzed by the treatment they received rather than how they were allocated.

The analysis of post-treatment assessments of secondary outcomes consisted of linear regressions adjusting for age, gender, treatment allocation, NFC, personal relevance, physical activity, and prior exposure. Models for physical activity determinants (i.e., intensions, attitudes, and perceived behavioral control) also adjusted for pre-treatment scores. Again, interactions between NFC and both treatment allocation and personal relevance were explored.

Analyses were performed in R (version 3.6.3) using package *glmmTMB* for the negative binomial mixed effects modeling. Significance was set at a threshold of 0.05 (two-sided).

## Results

### Participant Flow

**Fig. 3:**
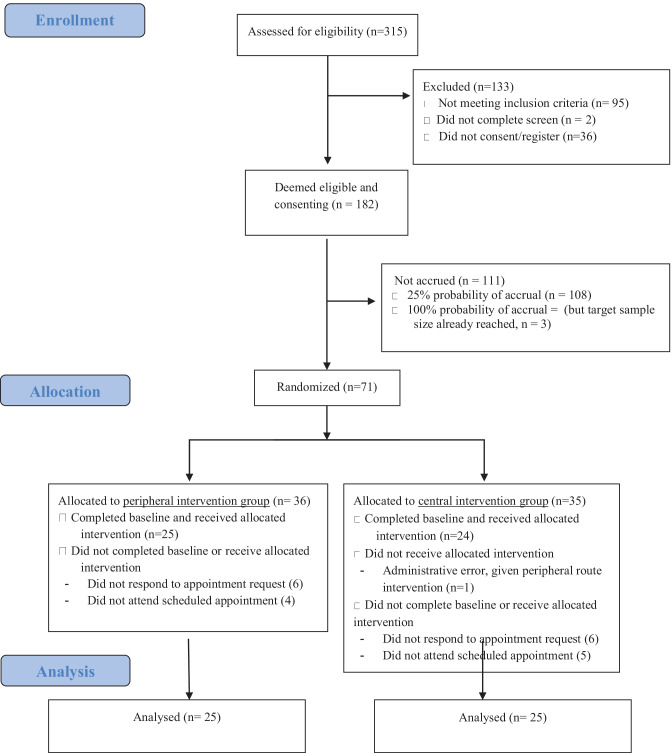
CONSORT flowchart presenting screening, accrual, randomization, and exclusion details

Participant Flow is summarized in Fig. 3. In total, out of 315 participants screened, 71 individuals were invited to participate and randomized prior to completing baseline assessments (based on pre-specified randomization protocol). Of those, 50 participants attended their study appointment (25 in each group). All data collection occurred at the study appointment.

### Participant Characteristics

Participants ranged in age from 18 to 55, with a median age of 21. Baseline physical activity levels were higher than expected (24% reported participating in 150 min or more per week of moderate to vigorous physical activity completed across at least 5 days of the week); however, physical activity habit strength (assessing the self-reported automaticity of physical activity) was low among all participants. NFC scores ranged from 21 to 92 (possible range is 0–108) with a mean of 64.1 (SD = 14.6). Participant characteristics were well-balanced across study conditions, with the exception of gender (12% in the peripheral route group as compared 42% in the central route group; see Table 1).

**Table 1:** Participant characteristics

	**Peripheral (*n* = 25)**	**Central (*n* = 25)**	**Total (*n* = 50)**
Male gender, *n* (%)	3 (12%)	10 (40%)	13 (26%)
Age, median (range)	20 (18–55)	21 (18–53)	21 (18–55)
Weekly income, *n* (%)			
≤ $1170	10 (40%)	9 (36%)	19 (38%)
> $1170	7 (28%)	11 (44%)	18 (36%)
Prefer not to answer	8 (32%)	5 (20%)	13 (26%)
Employment, *n* (%)			
Unemployed	2 (8%)	6 (24%)	8 (16%)
Casual	5 (20%)	2 (8%)	7 (14%)
Part-time	4 (16%)	4 (16%)	8 (16%)
Full-time	1 (4%)	2 (8%)	3 (6%)
Student	13 (52%)	11 (44%)	24 (48%)
Highest Education, *n* (%)			
High school	15 (60%)	11 (44%)	26 (52%)
Diploma	0 (0%)	5 (20%)	5 (10%)
University degree	10 (40%)	9 (36%)	19 (38%)
Relationship, *n* (%)			
Single	12 (48%)	11 (44%)	23 (46%)
In a relationship	11 (44%)	11 (44%)	22 (44%)
Married/de facto	2 (8%)	3 (12%)	5 (10%)
BMI, mean (SD)	24.2 (5.6)	24.0 (4.5)	24.1 (5.0)
Minutes/week moderate-vigorous physical activity, mean (SD)	212 (378)	143 (152)	178 (288)
Habit strength, mean (SD)	1.4 (1.3)	1.2 (1.0)	1.3 (1.1)
Prior exposure to physical activity information, *n* (%)			
A small amount	7 (28%)	10 (40%)	17 (34%)
A moderate amount	16 (64%)	13 (52%)	29 (58%)
A substantial amount	2 (8%)	2 (8%)	4 (8%)
Need for cognition	61.5 (13.1)	67.1 (15.7)	64.1 (14.6)
< 1 SD from mean, *n* (%)	5 (20%)	3 (12%)	8 (16%)
> 1SD from the mean, *n* (%)	3 (12%)	6 (24%)	9 (18%)

### Outcomes

#### Attention

##### Primary Outcome: Gaze Duration within AOIs

Due to the different quantity of text presented, the time participants spent viewing slides differed between intervention groups. The median AOI duration across all slides for the peripheral route intervention was 47 s (IQR = [39, 69]) compared to a median of 157 s (IQR = [122194]) in the central route group. Likewise, the median gaze time in AOIs per slide was shorter in the peripheral group (median = 3.1; IQR = [1.1, 4.9]) compared to the central route (median = 13.1; IQR = [7.4, 19.1]).

**Fig. 4:**
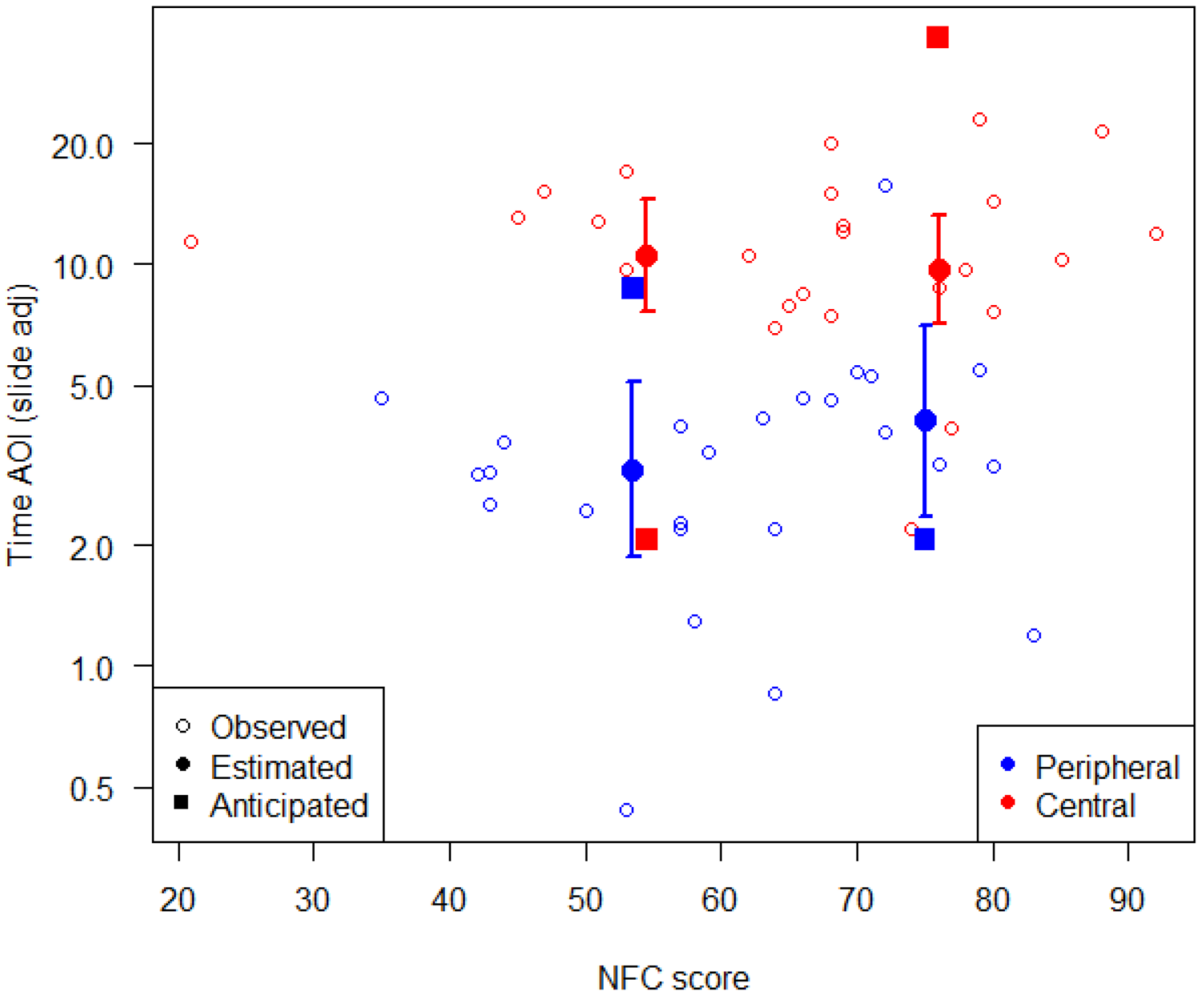
Interaction between need for cognition and group allocation on AOI gaze duration (adjusted for differences between slides). The point estimates and 95% confidence intervals are for the observed 25th and 75th need for cognition quantiles and the squares indicate the effect estimates used in the power calculation

The multivariable mixed-effects negative binomial regression for AOI gaze duration provided no evidence for associations with age (*p* = 0.41), gender (*p* = 0.77), NFC (*p* = 0.63), prior exposure to messages (*p* = 0.25) nor personal relevance (*p* = 0.08). There was weak evidence that individuals with higher physical activity levels exhibited reduced AOI times (β = 0.86, 95% CI = [0.73, 1.00], *p* = 0.05; see Table 2). With regard to the primary aim, no evidence was found for a matched-mismatched interaction between NFC and allocation (β = 0.78, 95% CI = [0.57, 1.07], *p* = 0.12), with the point estimate for the interaction being in the incorrect direction to what originally anticipated (see Fig. 4). The sensitivity analysis resulted in no qualitative differences for these conclusions, though notably the *p*-value for perceived personal relevance increased to *p* = 0.3 (see Supplementary Material 3). In regard to the secondary aim, there was no evidence of an interaction between personal relevance and NFC on the primary outcome (*p* = 0.67).

**Table 2:** Mixed effects regression models of the primary outcome, AOI gaze duration, and AOI ratio; (A) without and (B) with a pairwise interaction between need for cognition and intervention group

**AOI gaze duration**		**No interaction (A)**		**Interaction (B)**	
	**SD**	**Est [95% CI]**	***p*-value**	**Est [95% CI]**	***p*-value**
Intercept		3.48 [2.17, 5.59]	< 0.001	3.70 [2.31, 5.92]	< 0.001
NFC	14.3	1.04 [0.88, 1.23]	0.63	1.21 [0.94, 1.56]	0.13
Allocation (Cen vs Per)		2.96 [1.75, 5.01]	< 0.001	2.88 [1.72, 4.84]	< 0.001
Age	8.6	1.07 [0.91, 1.25]	0.41	1.06 [0.91, 1.24]	0.42
Gender (F v M)		0.95 [0.66, 1.37]	0.77	0.92 [0.64, 1.32]	0.65
MVPA^a^	296	0.86 [0.73, 1.00]	0.05	0.84 [0.72, 0.98]	0.03
Prior message exposure	0.796	0.92 [0.79, 1.07]	0.25	0.94 [0.81, 1.09]	0.37
Relevance	3.63	1.15 [0.98, 1.35]	0.08	1.17 [1.00, 1.37]	0.05
NFC × allocation				0.78 [0.57, 1.07]	0.12

**AOI ratio**		**No interaction (A)**		**Interaction (B)**	
	**SD**	**Est [95% CI]**	***p*-value**	**Est [95% CI]**	***p*-value**
Intercept		0.57 [0.48, 0.66]	< 0.001	0.59 [0.50, 0.68]	< 0.001
NFC score	14.3	0.00 [− 0.03, 0.03]	0.92	0.03 [− 0.02, 0.08]	0.19
Allocation (Cen vs Per)		0.10 [0.01, 0.20]	0.03	0.10 [0.01, 0.20]	0.03
Age	8.6	0.02 [− 0.01, 0.05]	0.2	0.02 [− 0.01, 0.05]	0.2
Gender (F v M)		0.00 [− 0.07, 0.07]	0.97	− 0.01 [− 0.08, 0.06]	0.82
MVPA^a^	296	− 0.02 [− 0.05, 0.01]	0.19	− 0.02 [− 0.05, 0.01]	0.12
Prior message exposure	0.8	− 0.01 [− 0.04, 0.01]	0.31	− 0.01 [− 0.04, 0.02]	0.45
Relevance	3.63	0.03 [0.00, 0.06]	0.08	0.03 [0.00, 0.06]	0.04
NFC × allocation				− 0.05 [− 0.11, 0.01]	0.1

^a^*MVPA*, moderate to vigorous physical activity reported at baseline, with vigorous activity minutes weighted by two

##### AOI Ratio

For AOI ratio, results were similar to those described above for AOI gaze duration, except that physical activity was not observed to be significantly associated with this outcome (*p* = 0.19; see Table 2).

##### Heatmaps

Differences in attention based on NFC (aim 1) were explored further by observing Heatmap data. Among those allocated to the central route group, those with high NFC appeared to spread their attention across the available information more evenly, whereas those with low NFC seemed to focus more attentively on key points (e.g., headings, dot-points; see Supplementary Material 4). Differences in gaze between those with high and low NFC appeared less pronounced on the heatmaps among individuals viewing peripheral route materials, though attention to written text did seem higher overall in those with a higher NFC. As noted, these differences did not translate into detectable differences in gaze duration.

#### Physical Activity Determinants

**Fig. 5:**
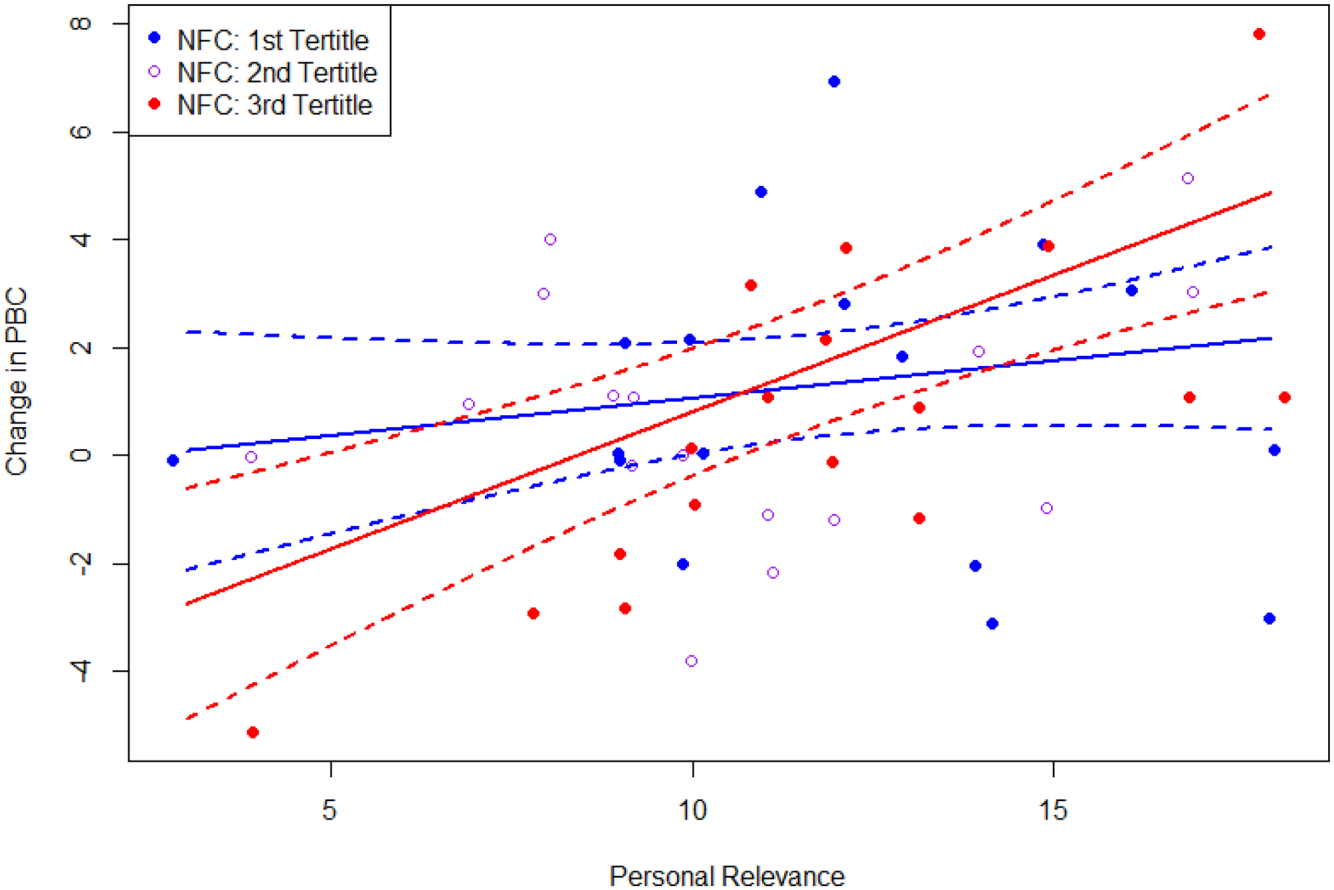
Interaction between personal relevance and NFC on change in perceived behavioral control

Physical activity intentions, attitudes, and perceived behavioral control scores were positive in both groups, with no between-group differences observed after adjusting for baseline (Table 3). The matched-mismatched interaction between NFC and allocation was not statistically significant for any of the physical activity determinant outcomes (aim 1, see Supplementary Material 5). However, all three post-intervention physical activity determinants were positively associated with personal relevance (intentions *p* = 0.02; perceived behavioral control *p* = 0.003, and attitudes *p* < 0.001). A one unit increase in perceived relevance was associated with 0.25–0.40 unit increase in the physical activity determinants (intentions, attitudes, and perceived behavioral control, rated on scales ranging from 0–18 to 0–28). An interaction between personal relevance and NFC was observed for perceived behavioral control (aim 2, Table 3). In particular, for participants higher in NFC, an increase in relevance was associated with an increase in perceived behavioral control (Fig. 5). There were no other significant interactions between NFC and either treatment allocation or relevance observed for any of the other outcomes (all *p*s > 0.20).

**Table 3:** Linear regression models of the physical activity determinant outcomes, (A) without and (B) with a pairwise interaction between need for cognition and relevance (for perceived behavioral control only)

		**Intentions (A)**		**Attitudes (A)**		**PBC (A)**		**PBC (B)**	
		**Coef [95% CI]**	***p*-value**	**Coef [95% CI]**	***p*-value**	**Coef [95% CI]**	***p*-value**	**Coef [95% CI]**	***p*-value**
Intercept		− 0.1 [− 4.7, 4.5]	0.96	− 0.9 [− 4.9, 3.2]	0.68	− 1.6 [− 6.6, 3.4]	0.54	12.9 [3.1, 22.6]	0.01
Baseline		0.69 [0.53, 0.85]	< 0.001	0.63 [0.50, 0.76]	< 0.001	0.68 [0.47, 0.89]	< 0.001	0.74 [0.55, 0.93]	< 0.001
Allocation (Cen vs Per)		− 0.8 [− 2.2, 0.7]	0.31	− 0.1 [− 1.4, 1.1]	0.86	− 0.6 [− 2.1, 1.0]	0.48	− 0.4 [− 1.8, 1.0]	0.55
NFC		0.04 [− 0.01, 0.09]	0.13	0.03 [− 0.01, 0.08]	0.12	0.04 [− 0.01, 0.09]	0.17	− 0.19 [− 0.33, − 0.04]	0.01
Relevance		0.25 [0.06, 0.44]	0.02	0.40 [0.24, 0.56]	< 0.001	0.34 [0.13, 0.55]	0.003	− 0.81 [− 1.52, − 0.09]	0.03
Age		− 0.04 [− 0.12, 0.04]	0.36	− 0.05 [− 0.12, 0.02]	0.15	− 0.04 [− 0.12, 0.05]	0.39	− 0.06 [− 0.14, 0.02]	0.12
Gender F v M		− 0.06 [− 1.74, 1.62]	0.94	0.40 [− 1.09, 1.89]	0.60	0.89 [− 0.83, 2.60]	0.32	1.57 [− 0.03, 3.17]	0.06
PME^a^		0.37 [− 0.44, 1.18]	0.38	0.55 [− 0.15, 1.24]	0.13	0.18 [− 0.69, 1.05]	0.68	− 0.03 [− 0.82, 0.76]	0.95
MVPA^b^		0.0011 [− 0.0013, 0.0035]	0.38	0.0015 [− 0.0005, 0.0036]	0.16	0.0001 [− 0.0025, 0.0028]	0.91	0.0005 [− 0.0019, 0.0028]	0.69
NFC × relevance		–		–		–		0.018 [0.007, 0.028]	0.002

^a^*PME*, prior message exposure

^b^*MVPA*, moderate to vigorous physical activity reported at baseline, with vigorous activity minutes weighted by two

#### User Experience and Perceptions

Participants in both groups rated the user experience similarly (peripheral group mean 40.7 (SD 9.8); central group mean = 42.4 (SD 12.9)), with scores suggesting a moderately positive experience (overall mean = 41.5 (SD 11.3), out of a possible 70). Perceived message effectiveness was also rated moderately by both groups (overall mean = 5.4 out of 9, SD = 1.4), with no between-group differences observed (Table 4). In contrast, there was a significant effect of group on message informativeness, with participants allocated to the peripheral route group rating the materials lower than participants in the central route group. This is to be expected by design. The interaction effect between NFC and either treatment allocation (aim 1) or personal relevance (aim 2) on these outcomes was not significant (all *p*s > 0.10). Perceived personal relevance was found to be positively associated with all outcomes (*p* < 0.01; see Table 4). A one unit increase in perceived relevance (rated on a scale ranging from 0 to 18), was associated with a 0.16 unit increase in perceived message effectiveness (rated on scale ranging from 0 to 9) and informativeness (rated on scale ranging from 0 to 8), and a 1.4 unit increase in user experience (rated on a scale ranging from 0 to 72).

**Table 4:** Linear regression models of user experience and perception outcome

		**Message effectiveness**		**Message informativeness**		**User experience**	
		**Coef [95% CI]**	***p***	**Coef [95% CI]**	***p***	**Coef [95% CI]**	***p***
Intercept		3.3 [0.9, 5.7]	0.01	3.5 [1.7, 5.3]	< 0.001	8.0 [–11.1, 27.0]	0.42
Baseline							
Allocation	C v P	0.2 [–0.6, 0.9]	0.68	1.2 [0.6, 1.7]	< 0.001	0.9 [–5.2, 7.1]	0.76
NFC		0.00 [–0.03, 0.03]	0.97	–0.01 [–0.03, 0.00]	0.15	0.02 [–0.18, 0.23]	0.82
Relevance		0.16 [0.06, 0.26]	0.004	0.16 [0.08, 0.23]	< 0.001	1.38 [0.57, 2.20]	0.002
Age		0.03 [–0.01, 0.07]	0.17	0.01 [–0.02, 0.04]	0.49	0.27 [–0.07, 0.61]	0.13
Gender	F v M	0.34 [–0.53, 1.21]	0.45	0.66 [0.01, 1.30]	0.05	5.3 [–1.5, 12.2]	0.14
PME^*a*^		–0.30 [–0.74, 0.13]	0.18	–0.09 [–0.41, 0.24]	0.60	2.1 [–1.3, 5.6]	0.23
MVPA^*b*^		0.0003 [–0.0010, 0.0015]	0.70	–0.0004 [–0.0013, 0.0005]	0.41	–0.0021 [–0.0121, 0.0078]	0.68

^a^*PME*, prior message exposure

^b^*MVPA*, moderate to vigorous physical activity reported at baseline, with vigorous activity minutes weighted by tw

## Discussion

The primary aim of this study was to examine the moderating role of NFC on the processing of physical activity promotion materials optimized for either central or peripheral route processing. Contrary to our hypothesis, we found no evidence of matching effects, whereby better message processing outcomes are observed in individuals receiving materials more aligned with their NFC. There was some evidence, however, that NFC was associated with how information was processed more generally (i.e., regardless of group). While NFC was not found to be directly associated with any of the message processing outcomes assessed (e.g., attention in areas of interest, acceptability, changes in attitudes), inspection of the heatmaps suggested that those with highest NFC may have paid closer attention to textual information across both conditions and overall were more likely to look at all elements within the materials than those with the lowest need for cognition. This observation is consistent with the notion that those with a higher NFC enjoy cognitive tasks and those with a lower NFC are inclined to reduce cognitive load [17, 18]. Given the heatmap data, one possible explanation for our null finding regarding the matching hypotheses is that NFC influenced how persuasion occurred within each group, but not the extent of persuasion. If this is so, it implies that both sets of materials contained both peripheral and central route processing cues, and that it may be possible to develop messages that can persuade using both pathways simultaneously, and, as such, are appropriate for people of any need of cognition. This speculation requires further exploration. To facilitate this, it is recommended that in future studies different types of areas of interest are specified and coded based on cue type. This would allow for examination of within-group processing differences.

Our secondary aim was to explore the role of perceived personal relevance as an influence on the relationship between need for cognition and message processing. Perceived personal relevance was found to be positively associated with message processing outcomes across groups, though this was not statistically significant for the attention outcomes. Further, contrary to our hypothesis, a significant interaction between NFC and relevance was only observed for one of the message outcomes, perceived behavioral control, and this was not in the pattern expected. Rather than finding a more pronounced difference between those with a higher versus lower NFC at moderate levels of relevance as per [23], the greatest differences were observed at the upper and lower thresholds of relevance. It seems, therefore, that relevance had little impact on perceived behavioral control for those with a low NFC, but that it did have an impact for those with a high NFC. For those individuals, the more relevant the materials were perceived, the greater impact the materials had on perceived behavioral control. This finding may offer a possible explanation for the results observed by Conner et al. [21]. Perhaps personal relevance of the materials optimized for central route processing were low in perceived relevance, reducing the persuasive effect of matched materials for those with high NFC.

Taken together, these findings reinforce existing evidence that personal relevance is key to persuasion [38], and suggest that relevance may be most important when targeting those with a higher NFC. Whether this depends on the messaging type employed (central route versus peripheral route) remains unclear as it was not possible to meaningfully test a three-way interaction in our study in light of our sample size (formulated based on the primary aim).

This study presents several limitations that should be acknowledged. First, the majority of our sample were female and younger adults, with a median age of 21. The generalizability of our findings to less represented groups such as men and older adults is therefore unclear. Some participants (24%) also reported activity levels in line with national guidelines during the baseline assessment. As active participants are likely to have high baseline scores on determinants of exercise measures, this may have reduced power when examining changes in intention, attitudes, and perceived behavioral control beliefs due to ceiling effects. Our accrual protocol also resulted in the randomization of 21 individuals who did not participate in any study procedures, including the baseline assessment. This made an intention-to-treat analysis impossible and instead we have only conducted a complete case analysis, which may have introduced some selection bias. While it does appear that our accrual protocol performed well in terms of maximizing variance in NFC (compared to population norms [22]), randomization should have occurred after baseline assessment. It may have also been useful to include demographic factors into our accrual algorithm (e.g., eligible women have accrual probability of 0.6) to ensure a greater demographic heterogeneity. With these refinements, use of our recruitment protocol may be useful in future research to balance timely recruitment with the need for variance in key variables.

Second, our study did not include a behavioral outcome. It is unclear if the differences we saw in persuasion and user experience will actually translate into behavior change. Overall, our results should be considered hypothesis generating, with future studies testing the impact of message type, and the moderating role of NFC and personal relevance on behavior recommended. This study, however, is an important first step in understanding the moderators of physical activity message processing, which is critical to our capacity to better target physical activity promotion messages in the future. Key strengths of the study include the examination of multiple dimensions of message processing, the use of a strategic accrual protocol, exploration of NFC in continuous rather than binary form, the rigorous theory–based development of study stimuli, and mixed measures to assess outcomes.

Overall, the results of our study suggest that matching physical activity messages based on NFC may not be sufficient to increase message persuasiveness. This may be dependent on the types of cues that are present in the messages, the underlying determinants of behavior targeted, the number of times exposed, and other tailoring factors. Perceived personal relevance of materials does appear to be associated with greater change in physical activity determinants and may be more so the case among those with a higher need for cognition. These findings may be useful to consider when developing new physical activity promotion materials. In particular, where central route persuasion methods are utilized, strategies to increase the relevance of materials are recommended. This could include collaborative processes with intended end-users to identify and refine content, as well as intervention techniques designed to increase personalization (e.g., computer-tailoring and recommender systems). A greater focus on embedding persuasive peripheral cues into any developed physical activity promotion materials may also be beneficial. While further research is needed, it may be possible to design materials that can persuade via both the central and peripheral pathways. If so, such materials may be able to persuade a wider variety of audiences and may also be more effective than materials designed to persuade via a single pathway. Further research examining any possible synergistic or detrimental effects of targeting both pathways at once is recommended.

## Supplementary Information

Below is the link to the electronic supplementary material.

Supplementary file1 (DOCX 24288 KB)
