# Supplementary material for: Exploring the Interplay Between Message Format, Need for Cognition and Personal Relevance on Processing Messages About Physical Activity: a Two-Arm Randomized Experimental Trial
Source: Int J Behav Med. 2022 Jun 10;30(3):320–33. doi: 10.1007/s12529-022-10107-4 (PMC10167115; doi:10.1007/s12529-022-10107-4)
Supplement: Supplementary file 1 — Supplementary file1 (DOCX 24288 KB) [file 12529_2022_10107_MOESM1_ESM.docx]

Supplementary material 1. Correspondence between key intervention messages, determinants targeted and intervention strategies across conditions.

| **Key messages** | **Determinants targeted** | **Strategies** |
| --- | --- | --- |
| Exercise is for everyone | Experiential attitudes | Direct statement |
| Exercise has immediate benefits | Instrumental attitudes | Provide information on the immediate consequences of the behavior |
| There are two types of exercise recommended to obtain health benefits: aerobic and resistance-based | Instrumental attitudes | Provide information on behavior-health link  Direct statement, with examples |
| You can start small and ramp up overtime | Experiential attitudes  Perceived behavioral control | Encourage setting of graded tasks |
| To stay on track set goals, schedule it in, monitor progress and make it social | Perceived behavioral control | Encourage goal setting  Prompt self-monitoring of behavior  Prompt time management |
| Begin | Intentions | Direct statement |

Supplementary material 2: Overview of Theory-based Physical Activity Determinants Assessed pre and post exposure.

| Construct | Items | Response Options |
| --- | --- | --- |
| Intentions | 1. In the next two weeks, my goal is to be physically active 2. Over the next two weeks, I intend to engage in physical activity ____ times per week 3. I intend to engage in physical activity at least every other day over the next two weeks | 1. 7-point scale from 1-not at all to 7- everyday 2. Rated on a scale of 0-50 3. Rated on a 7-point scale from strongly disagree to strongly agree. |
| Attitudes | For me, engaging in regular physical activity over the next two weeks would be:   1. Harmful-Beneficial (instrumental) 2. Useless-Useful (instrumental) 3. Unenjoyable-enjoyable (affective) 4. Unpleasant-pleasant (affective) | 1-4: Bipolar adjective 7-point response scale |
| Perceived behavioral control | 1. In the next two weeks, doing physical activity if I really wanted to is under my control” 2. I am confident I could engage in physical activity in the next two weeks if I wanted to” 3. Engaging in regular physical activity over the next two weeks if I wanted to would be easy. | 1-4: Rated on a 7-point likert scale, ranging from strongly disagree to strongly agree. |

Supplementary material 3: Sensitivity analysis for the primary outcome excluding the patient with unusually long reading times and analysing individuals by the treatment they received; (A) without and (B) with a pairwise interaction between need for cognition and intervention group.

| **AOI Gaze Duration** | | **No Interaction (A)** | | **Interaction (B)** | |
| --- | --- | --- | --- | --- | --- |
|  | **SD** | **Est [95% CI]** | **p-value** | **Est [95% CI]** | **p-value** |
| Intercept |  | 2.58 [1.70, 3.90] | <0.001 | 2.66 [1.75, 4.04] | <0.001 |
| NFC | 14.5 | 1.01 [0.88, 1.15] | 0.91 | 1.08 [0.88, 1.33] | 0.47 |
| Allocation (Cen vs Per) | | 4.80 [2.96, 7.79] | <0.001 | 4.73 [2.92, 7.68] | <0.001 |
| Age | 8.67 | 1.11 [0.97, 1.26] | 0.11 | 1.11 [0.97, 1.26] | 0.11 |
| Gender (F v M) | | 1.04 [0.77, 1.40] | 0.78 | 1.03 [0.76, 1.38] | 0.87 |
| MVPA^1^ | 299 | 0.87 [0.77, 0.99] | 0.03 | 0.87 [0.76, 0.98] | 0.03 |
| Prior Message Exposure | 0.766 | 0.97 [0.86, 1.09] | 0.56 | 0.97 [0.86, 1.10] | 0.65 |
| Relevance | 3.63 | 1.07 [0.94, 1.22] | 0.3 | 1.08 [0.95, 1.23] | 0.24 |
| NFC x Allocation | |  |  | 0.90 [0.69, 1.16] | 0.39 |

*^1^MVPA = moderate to vigorous physical activity reported at baseline, with vigorous activity minutes weighted by two.*

| **CENTRAL ROUTE MATERIALS** | |
| --- | --- |
| HIGH NFC – TOP 3^rd^ NFC SCORES (M = 81.66) | LOW NFC – BOTTOM 3^rd^ NFC SCORES (M = 45) |
| 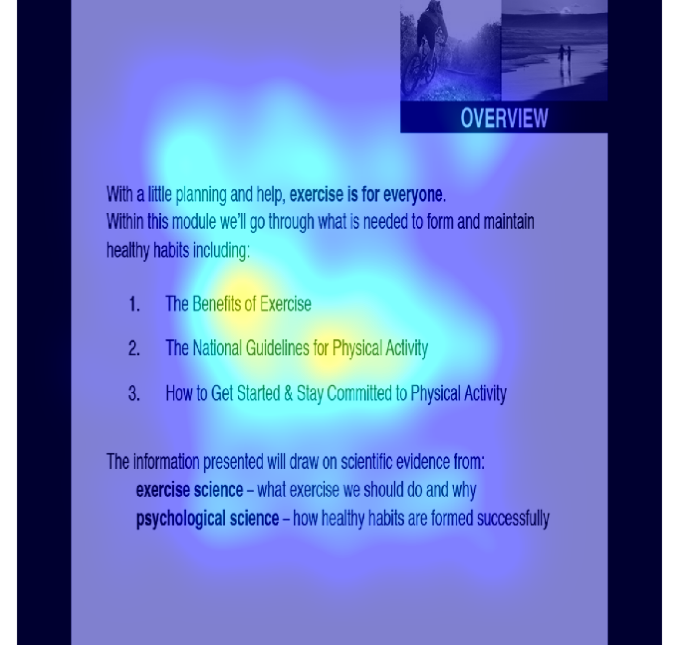 | 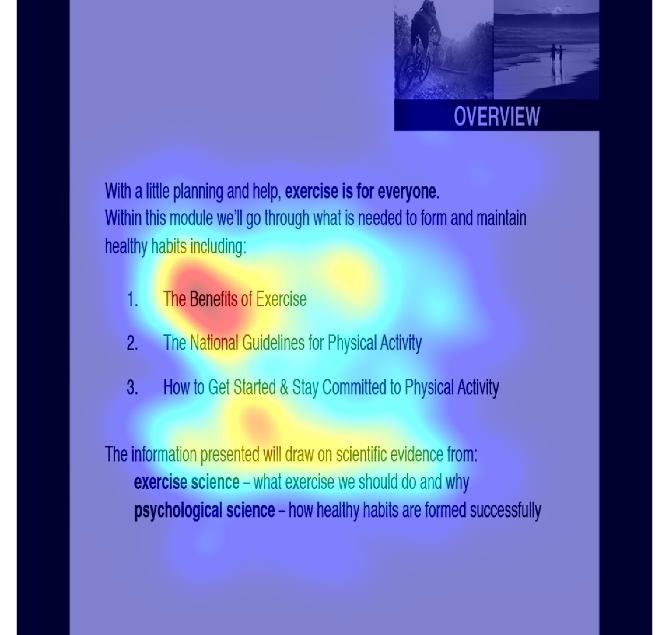 |
| 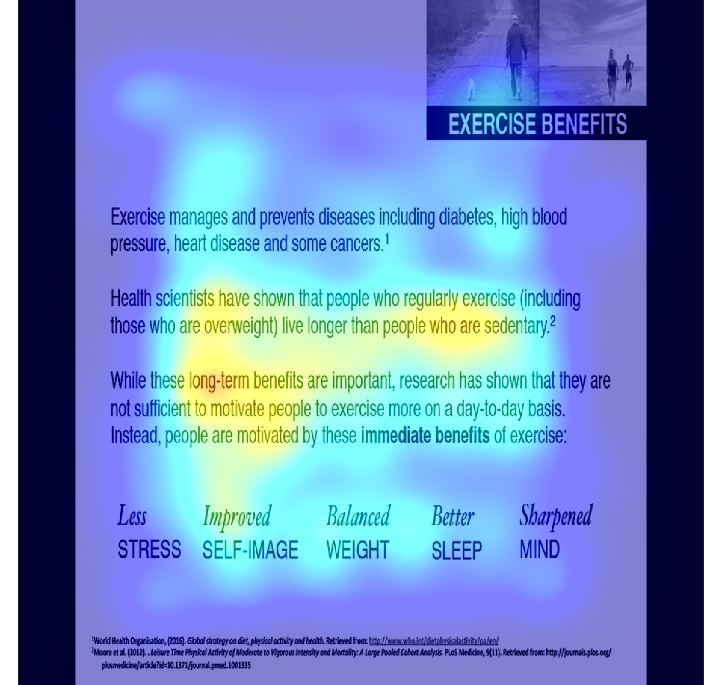 | 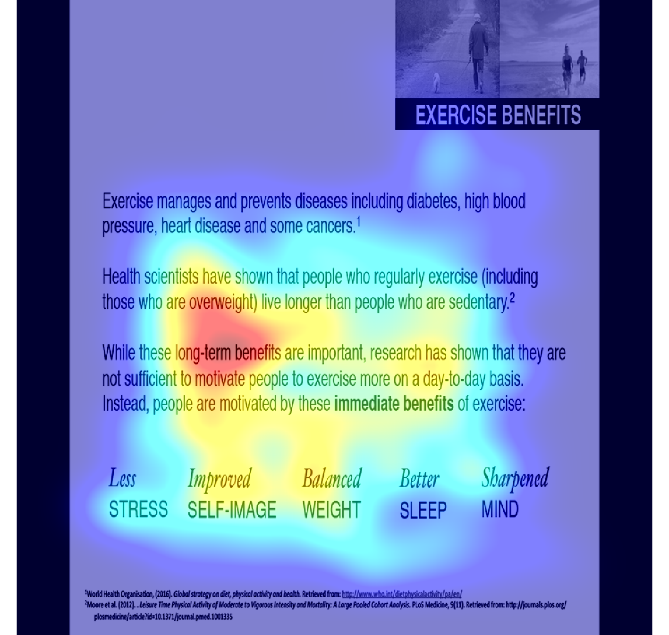 |
| 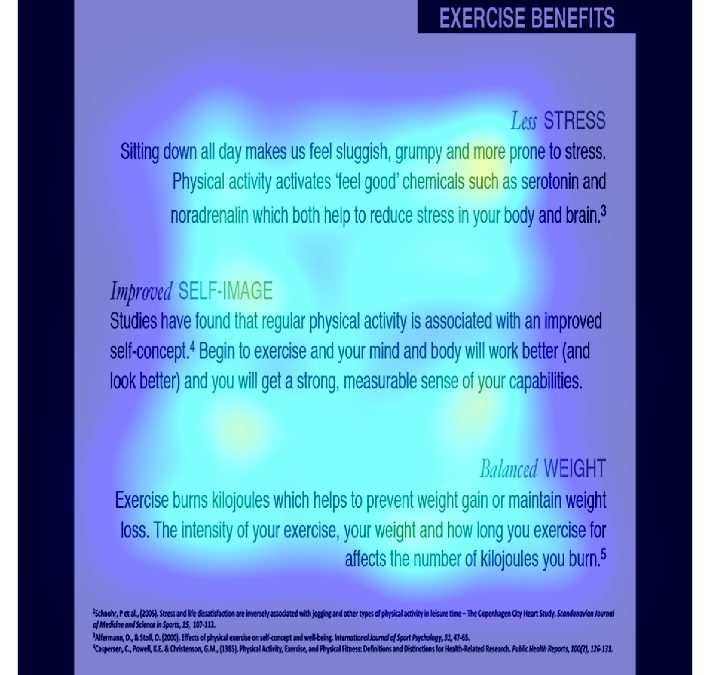 | 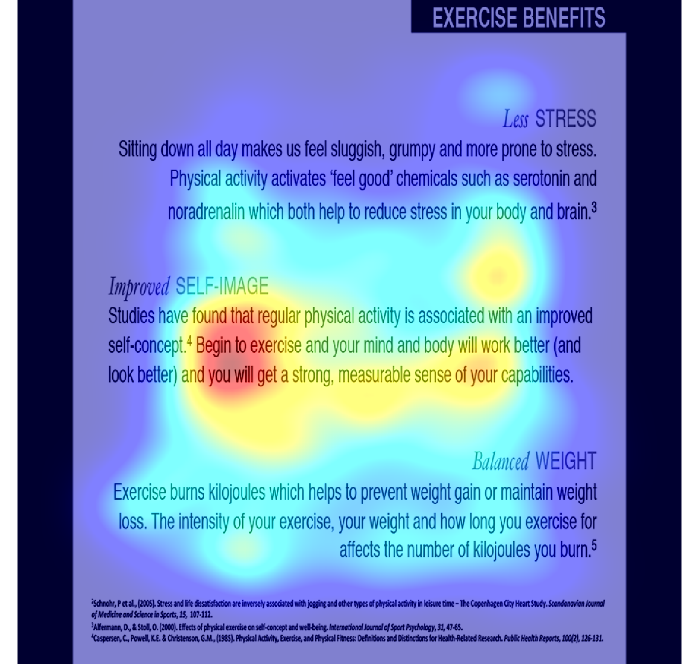 |
| 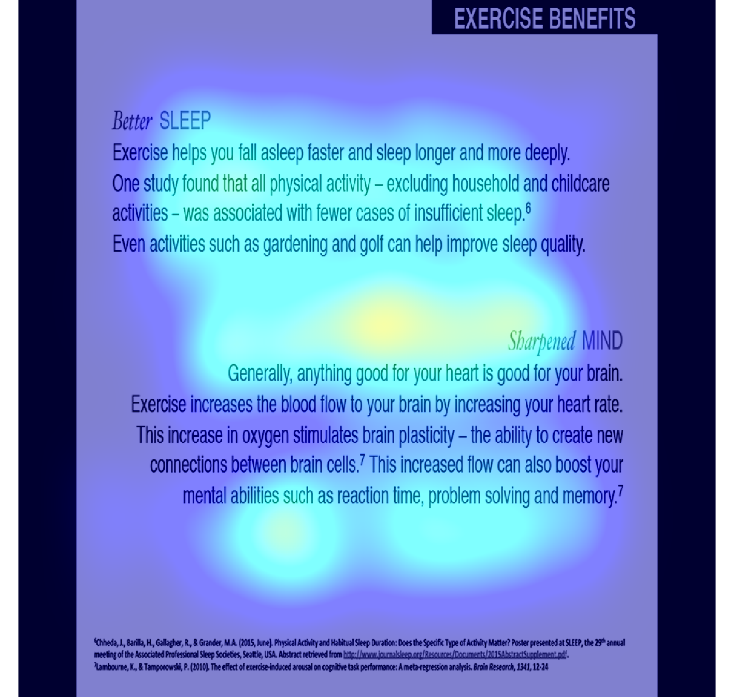 | 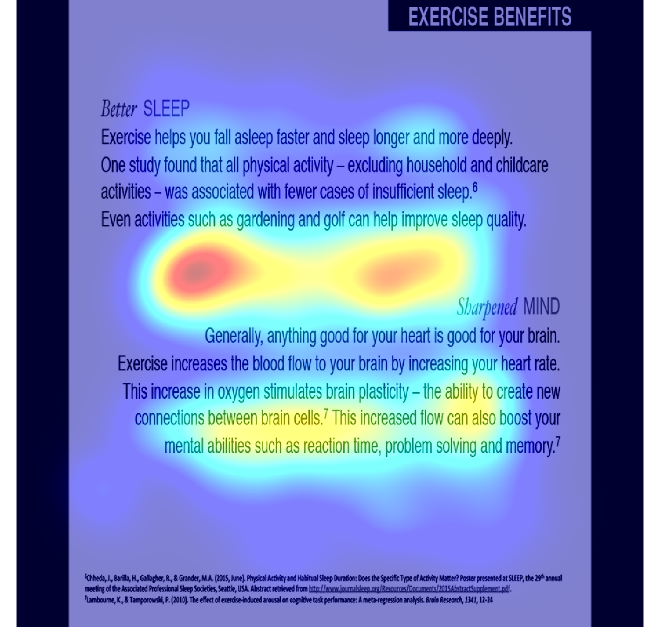 |
| 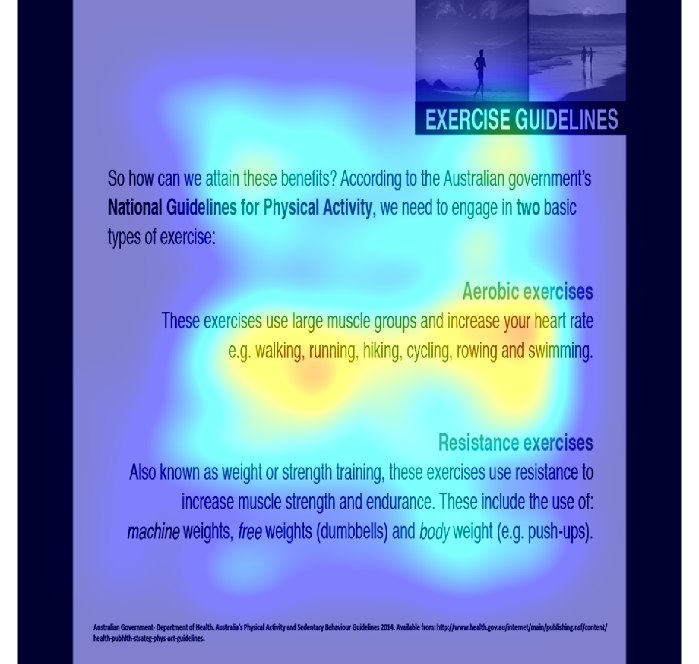 | 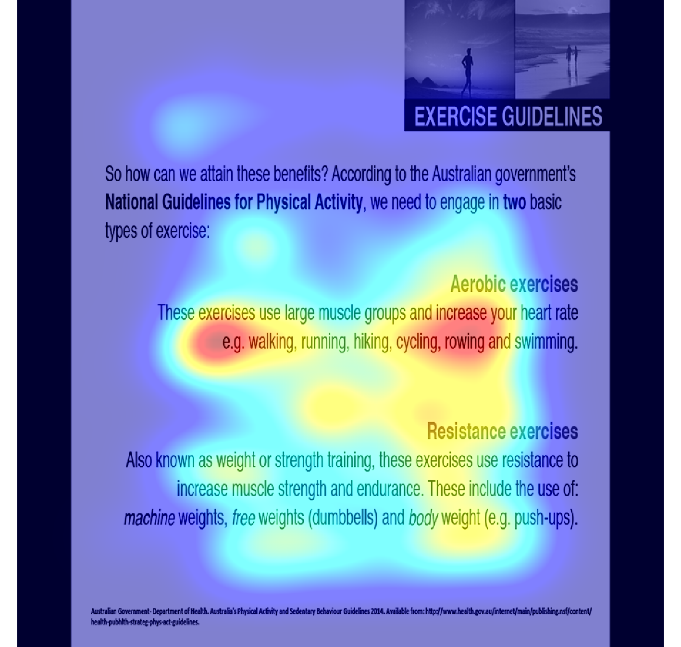 |
| 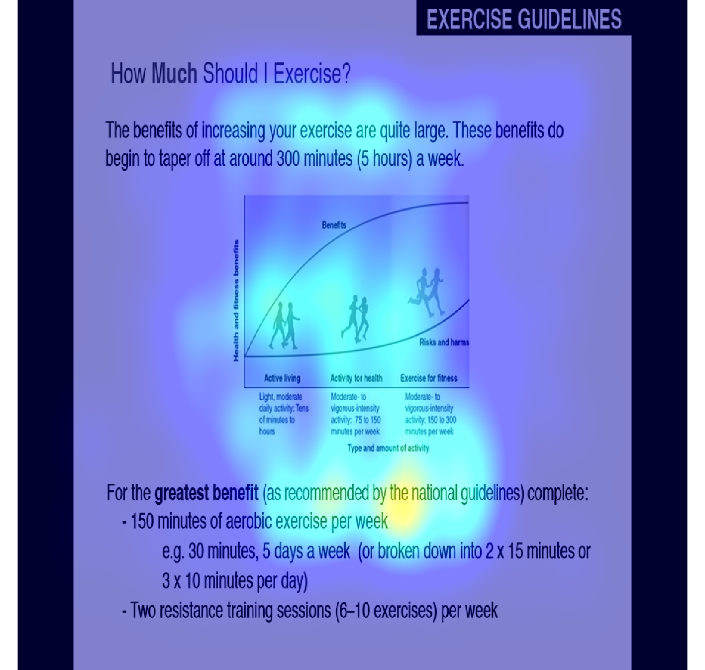 | 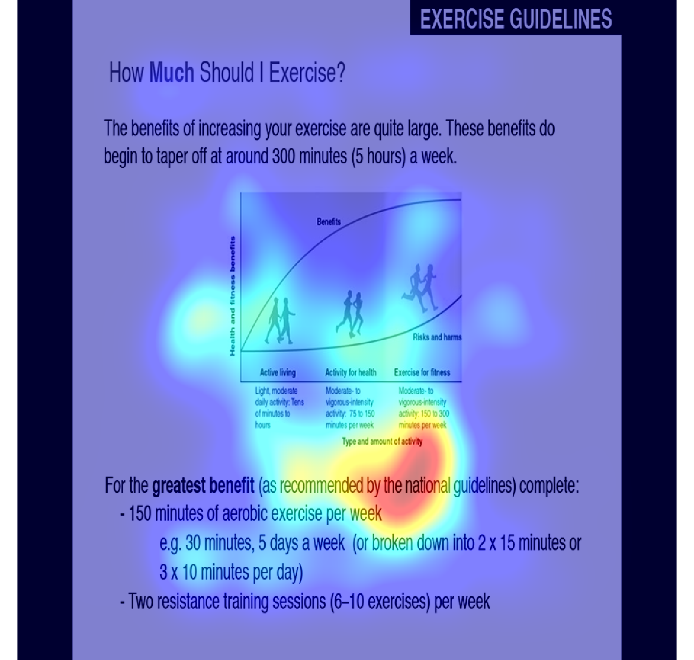 |
| 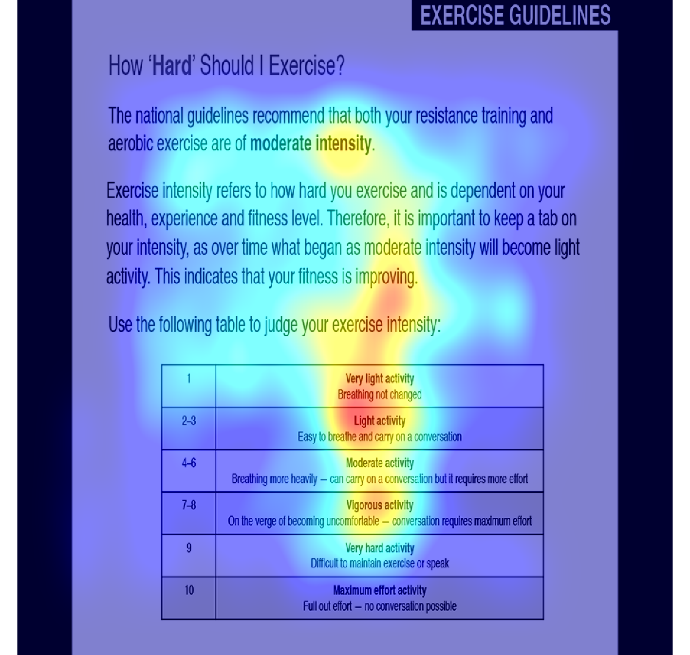 | 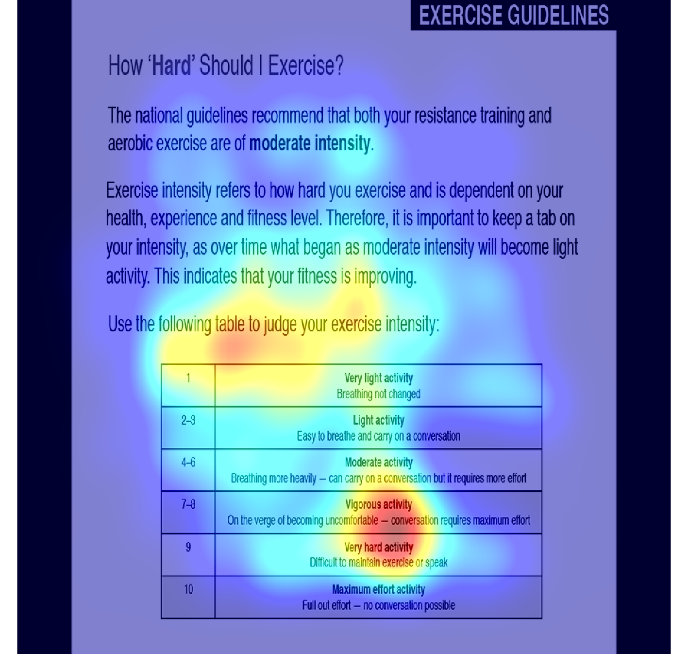 |
| 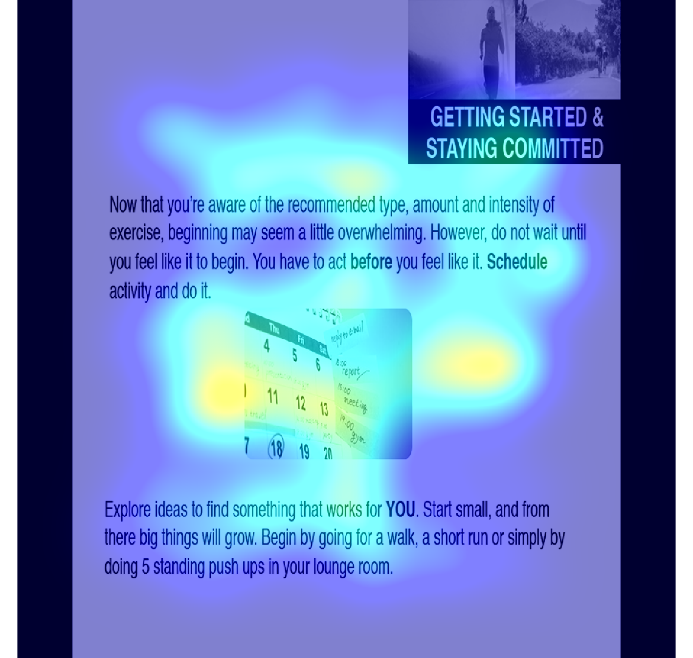 | 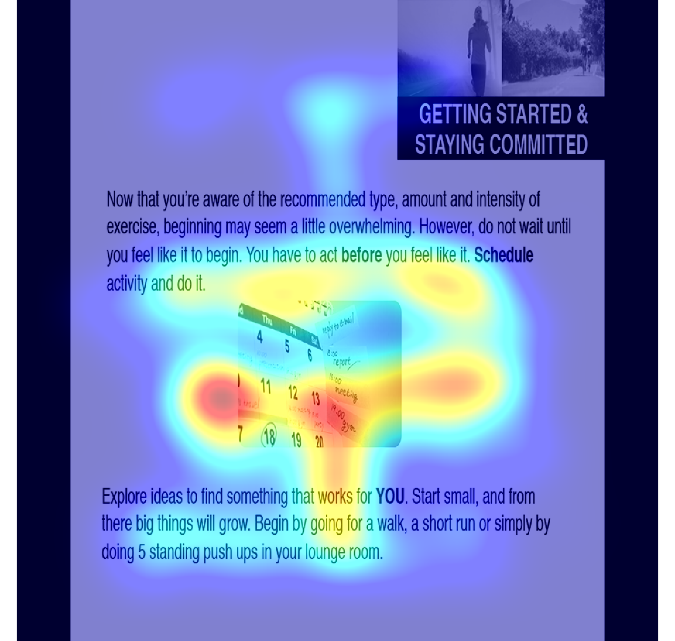 |
| 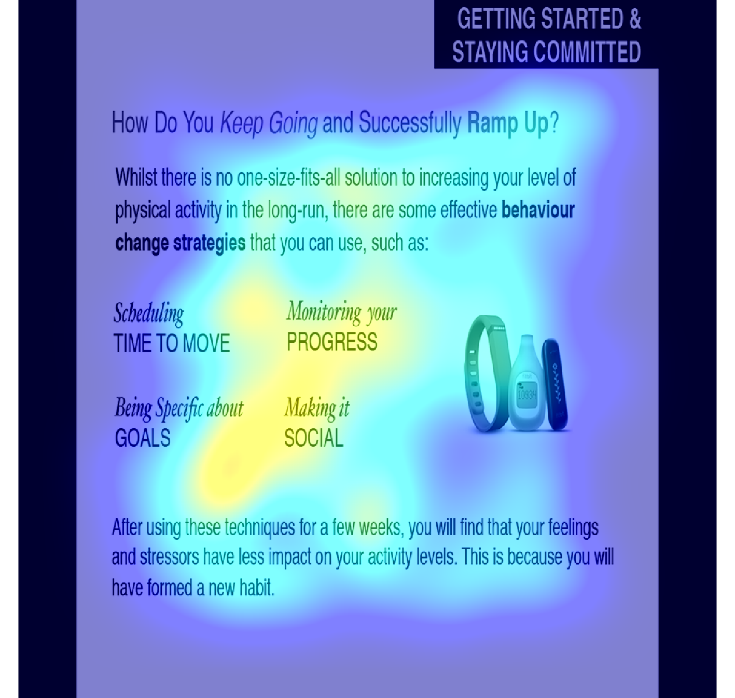 | 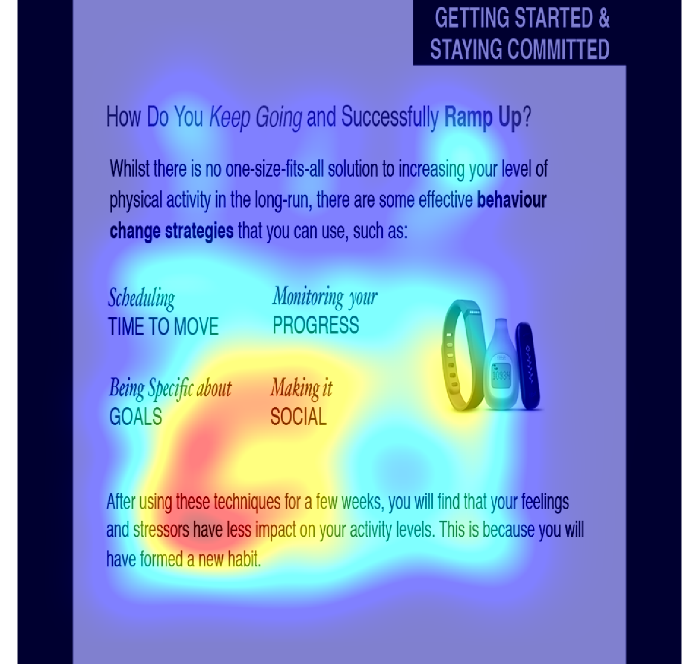 |
| 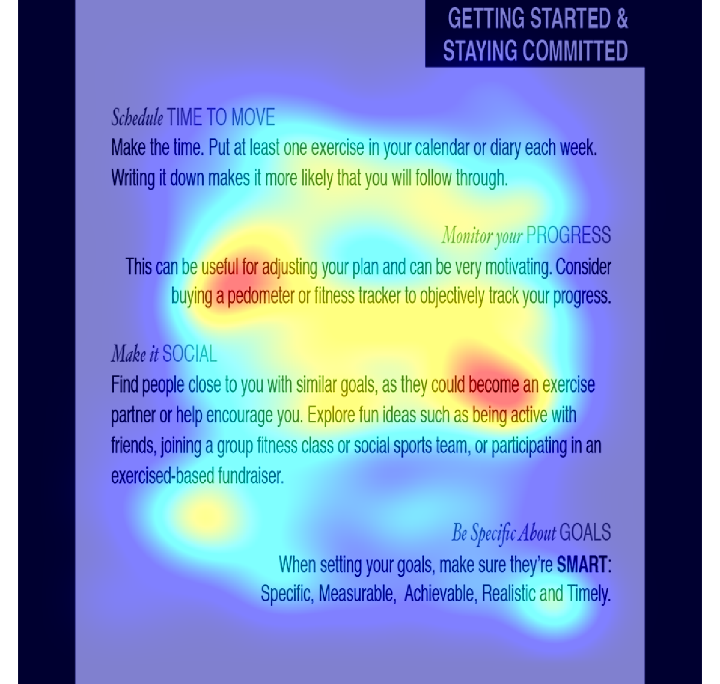 | 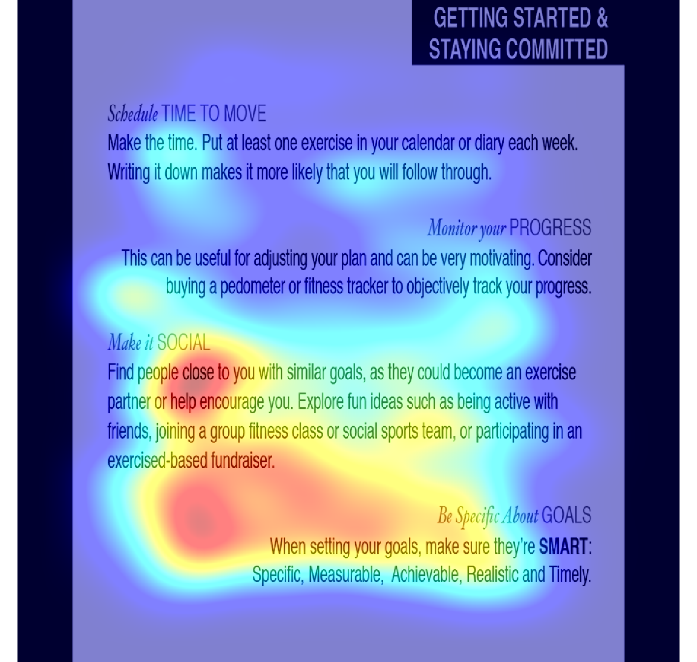 |
| 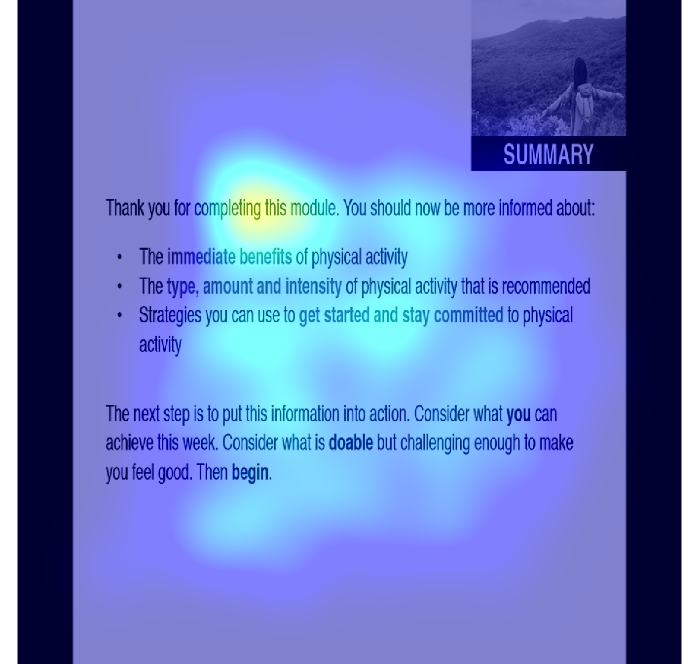 | 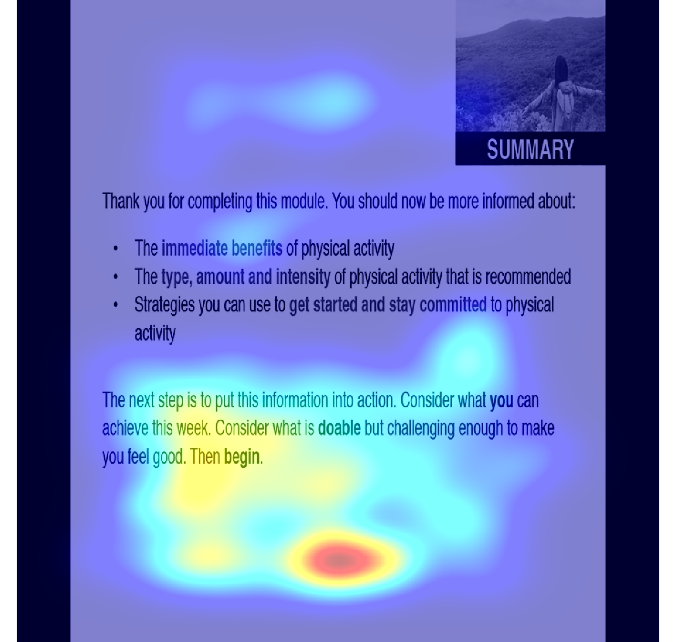 |
| **Peripheral ROUTE MATERIALS** | |
| HIGH NFC – TOP 3^rd^ NFC SCORES | LOW NFC – BOTTOM 3^rd^ NFC SCORES ) |
| 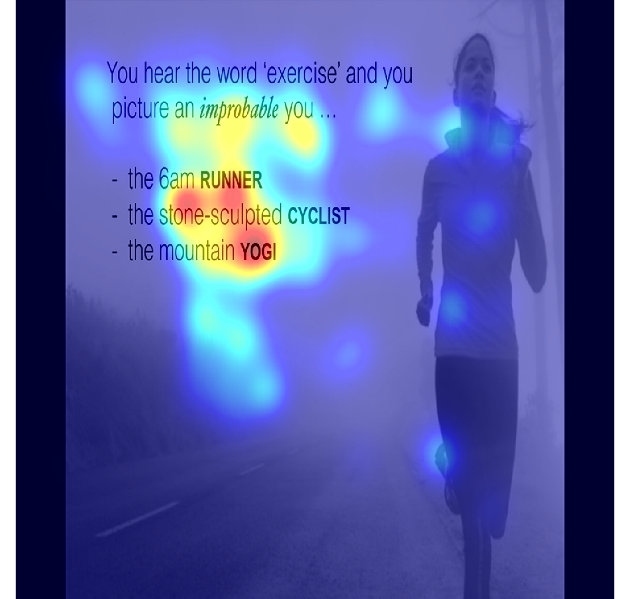 | 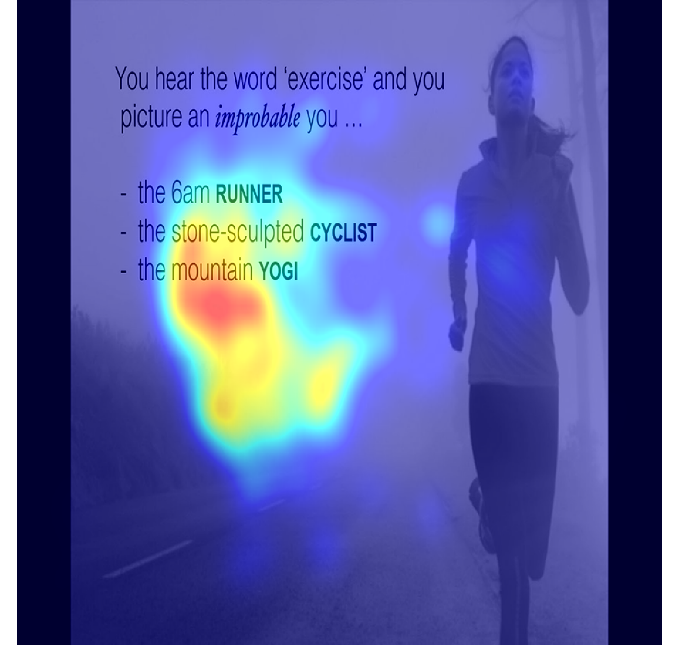 |
| 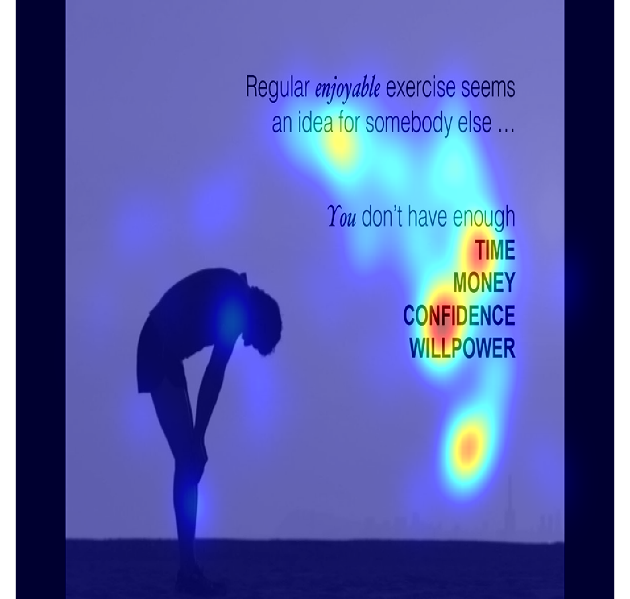 | 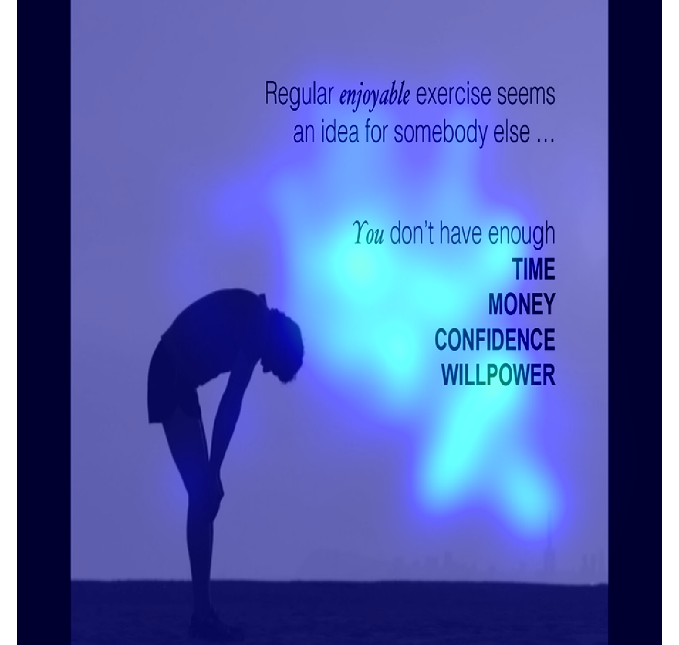 |
| 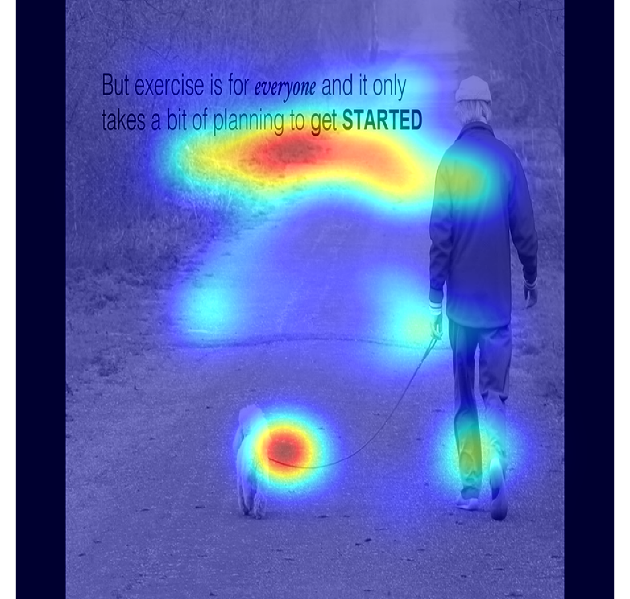 | 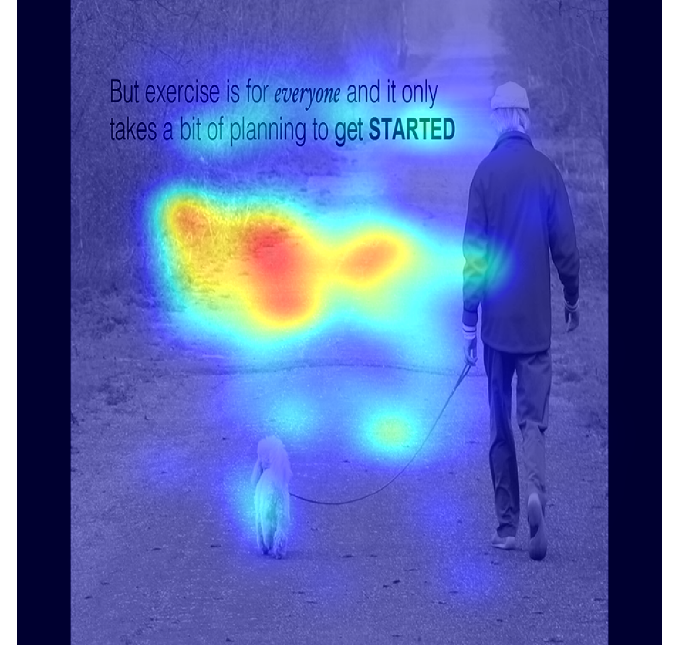 |
| 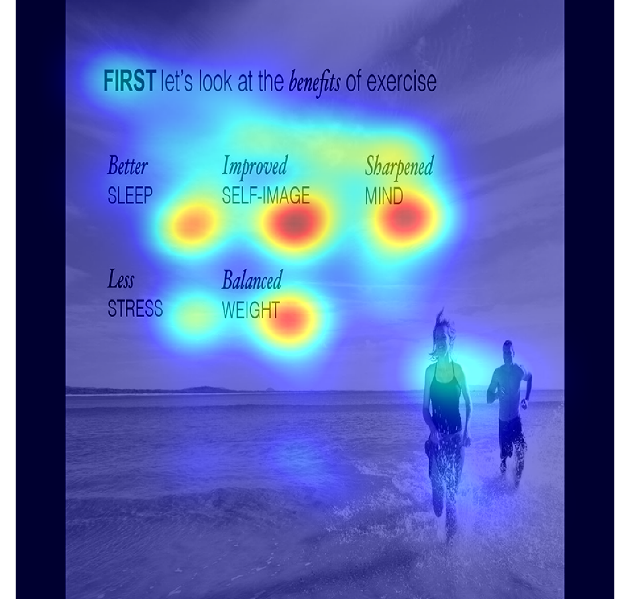 | 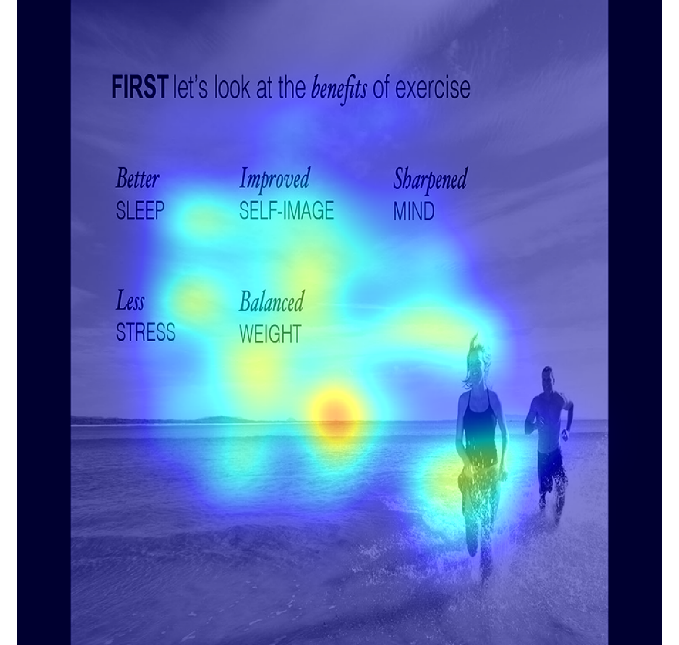 |
| 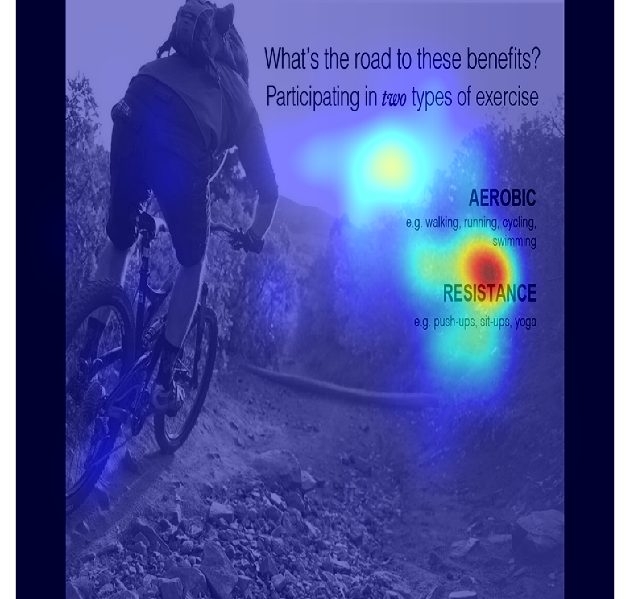 | 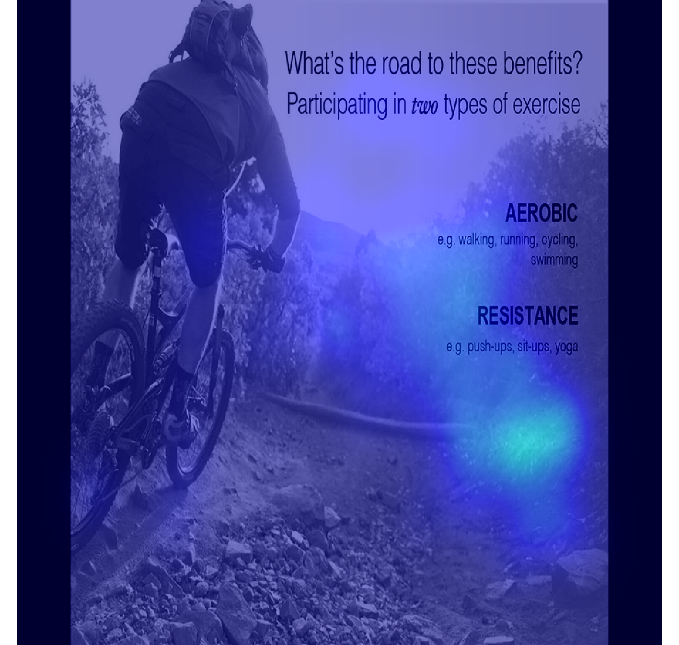 |
| 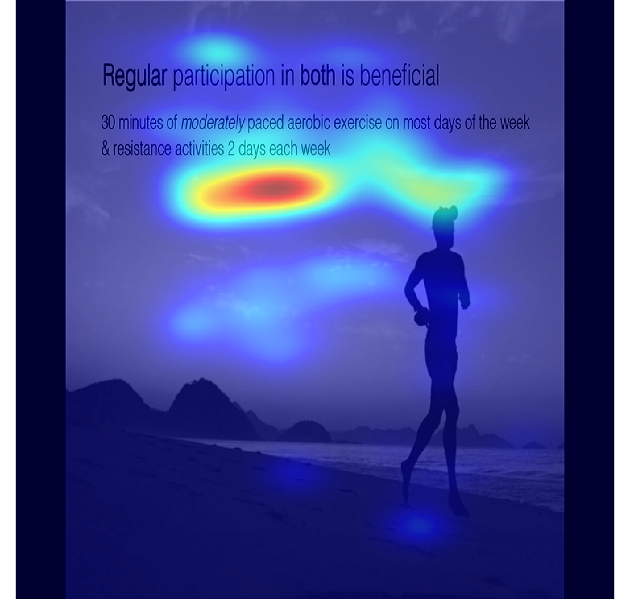 | 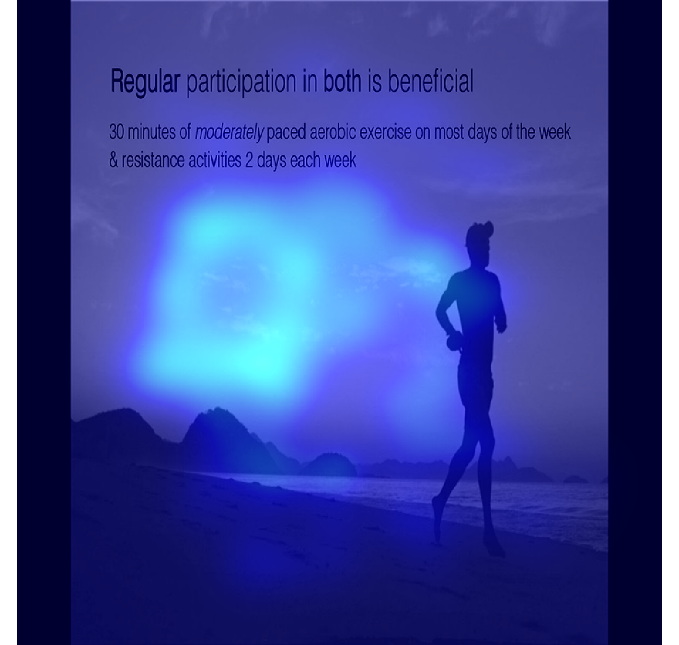 |
| 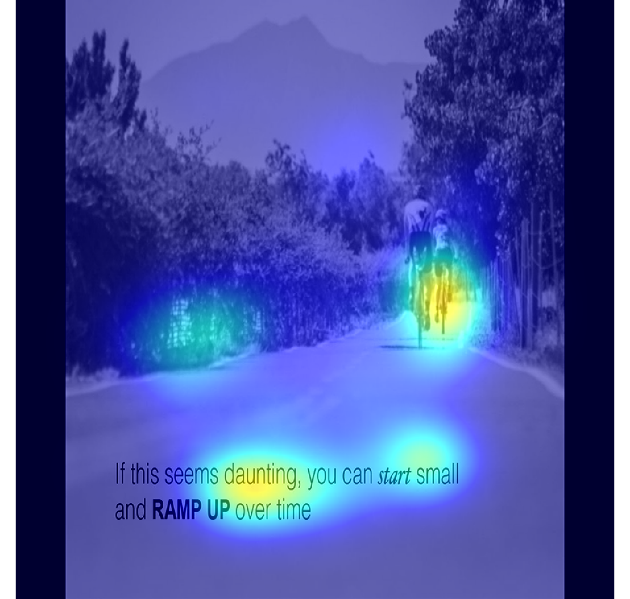 | 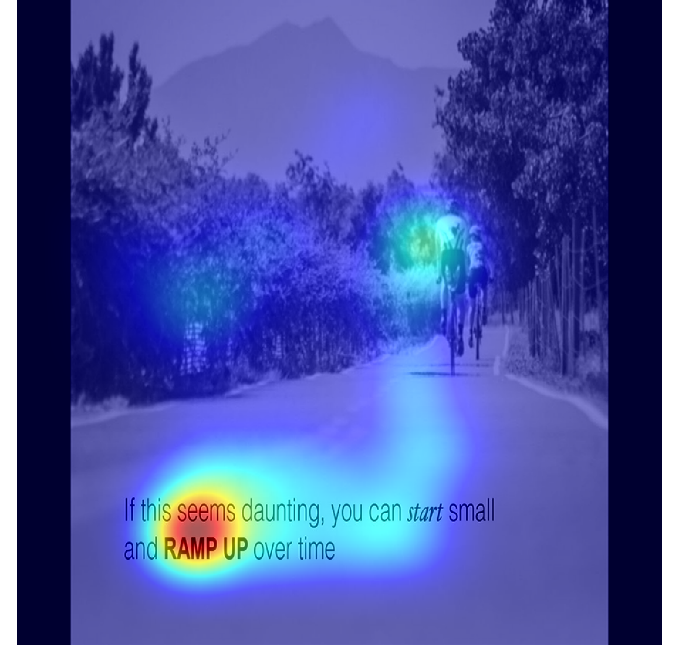 |
| 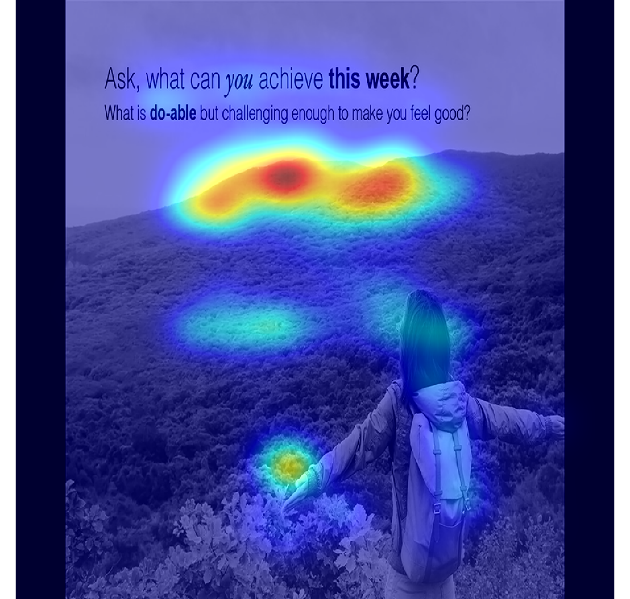 | 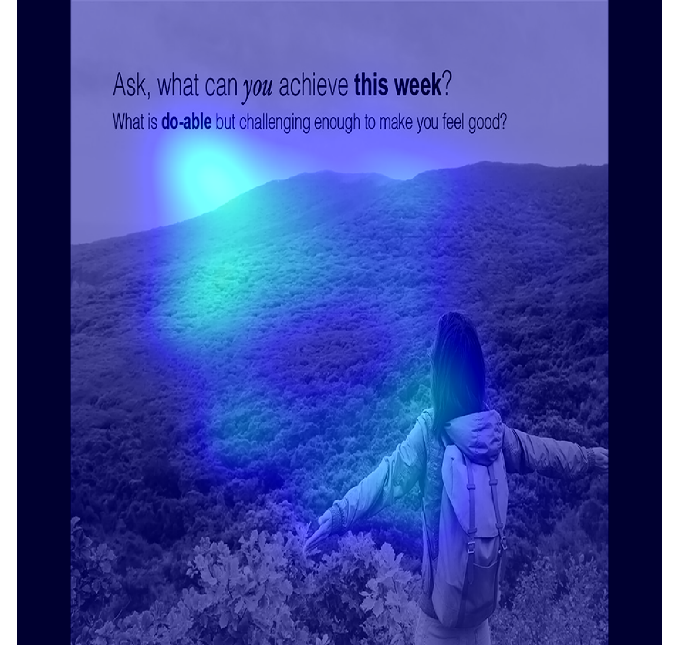 |
| 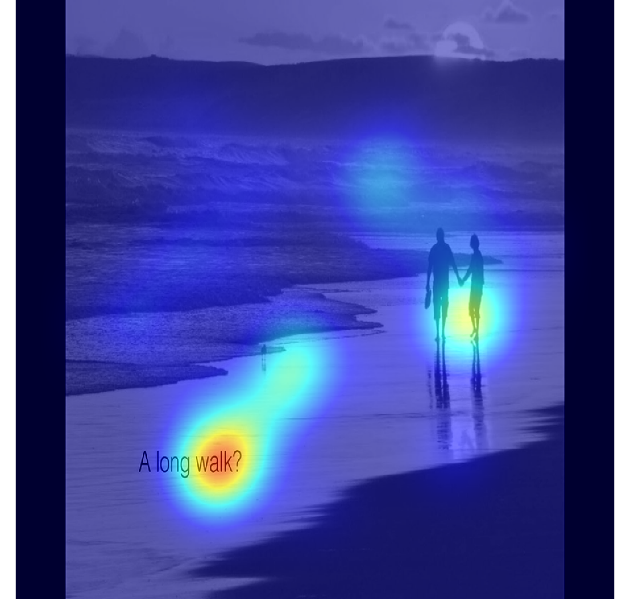 | 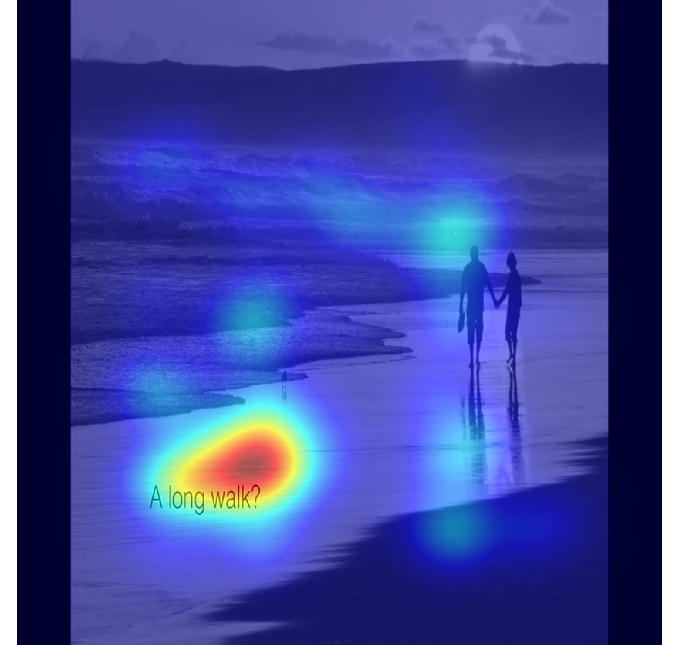 |
| 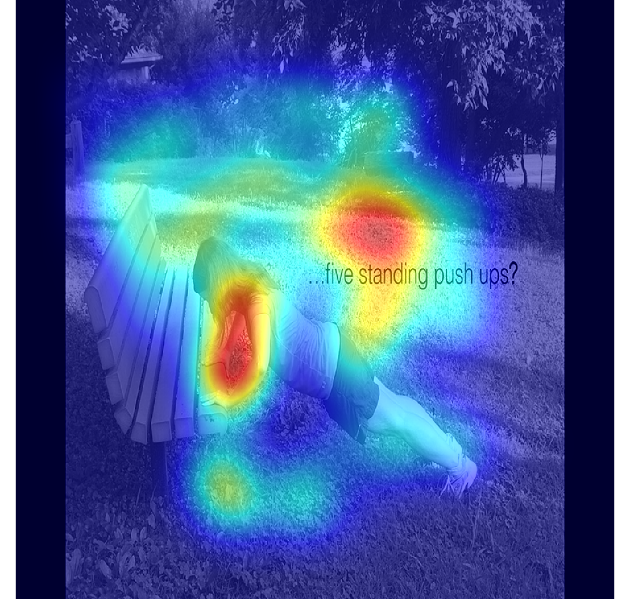 | 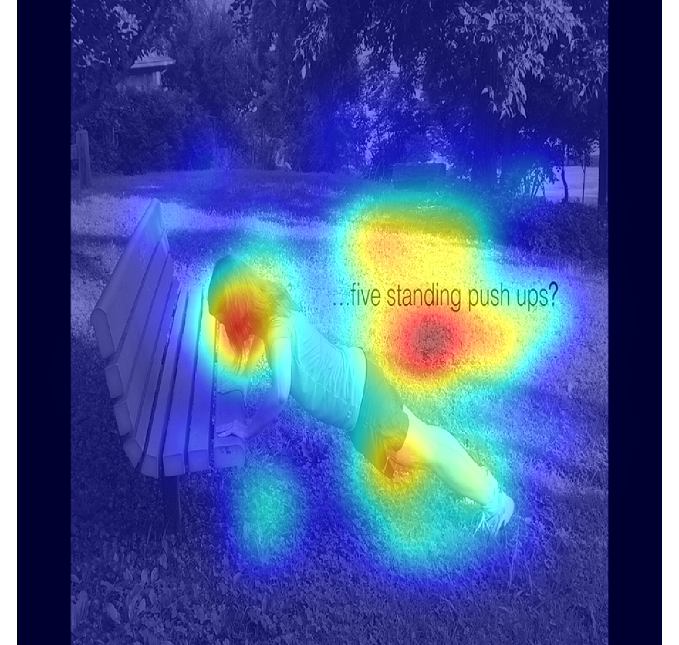 |
| 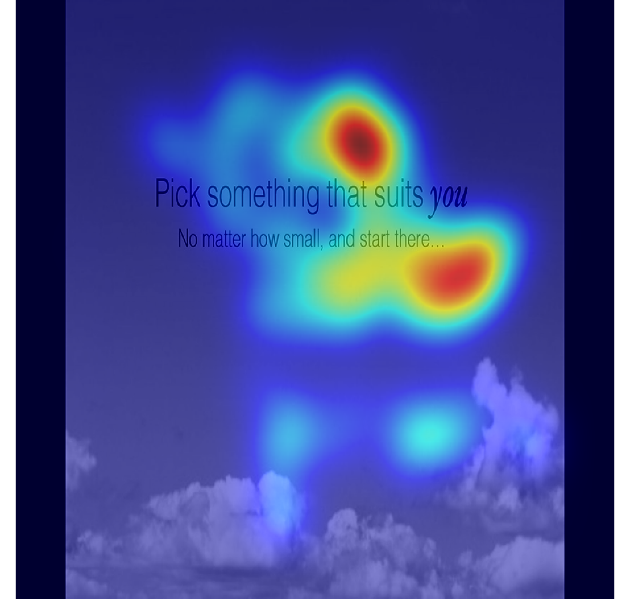 | 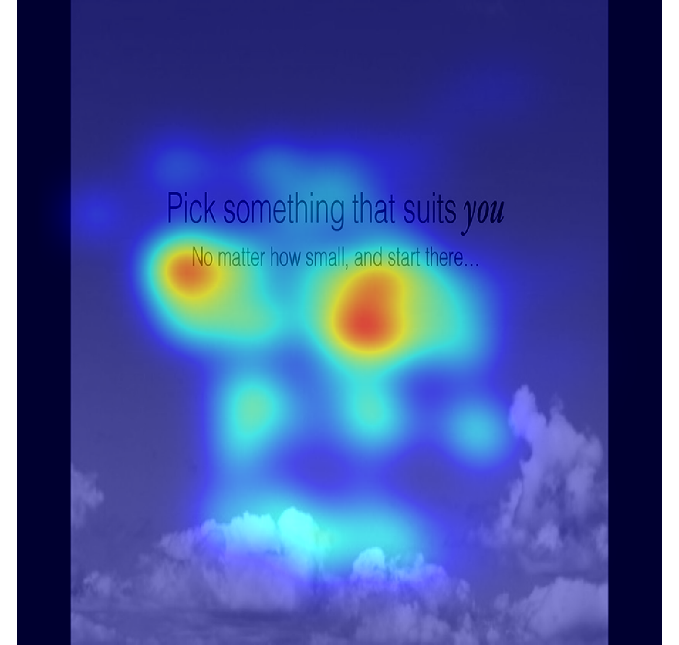 |
| 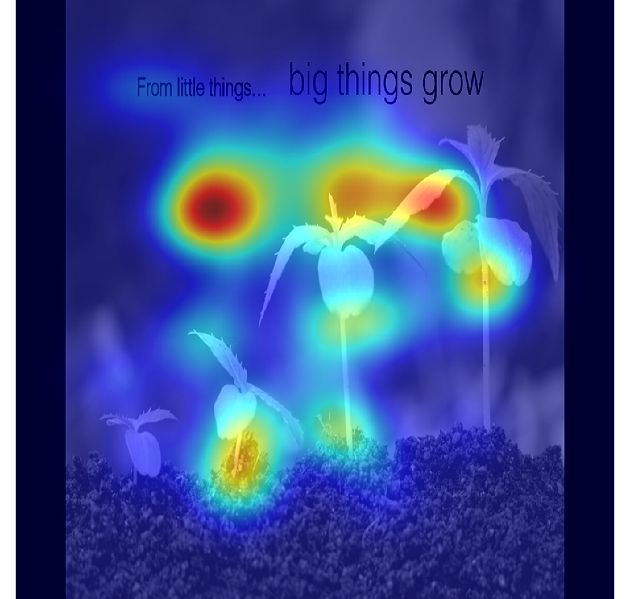 | 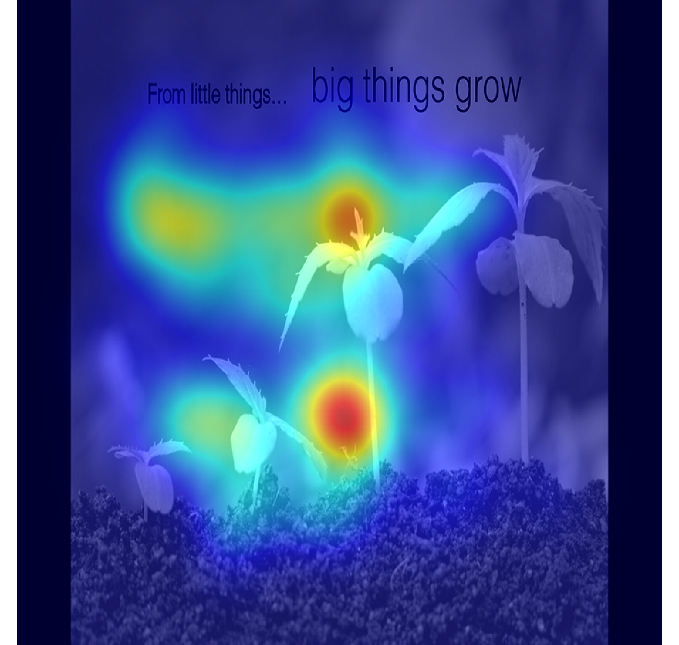 |
| 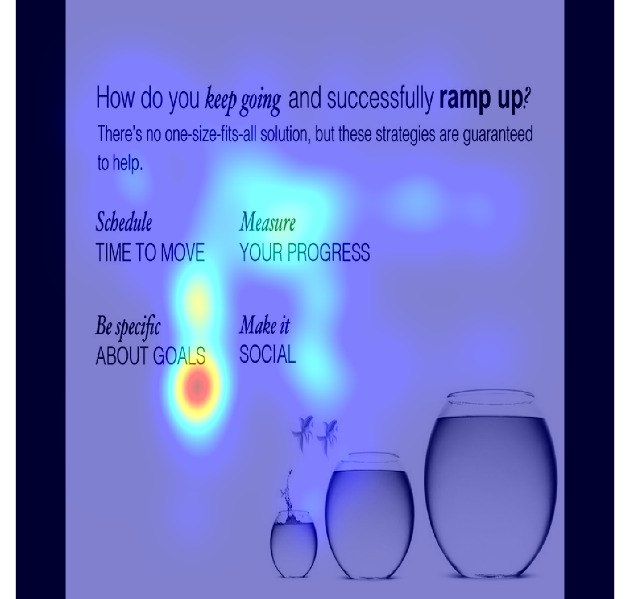 | 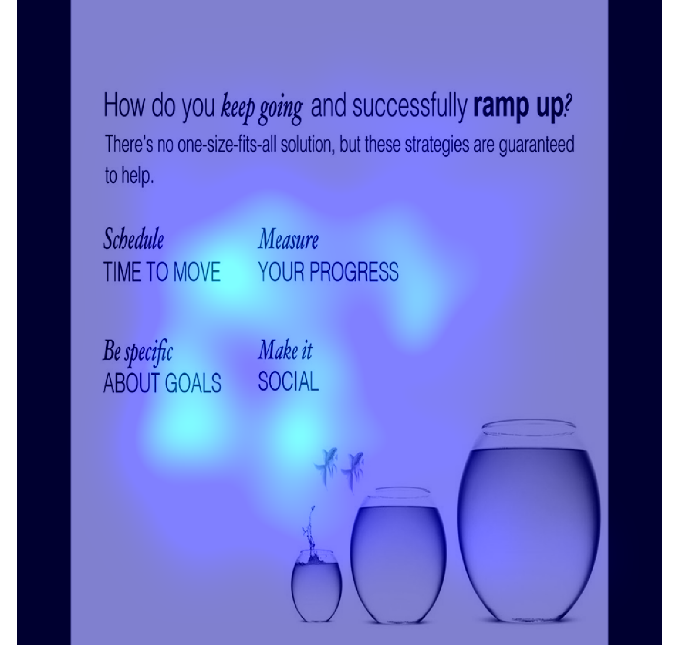 |
| 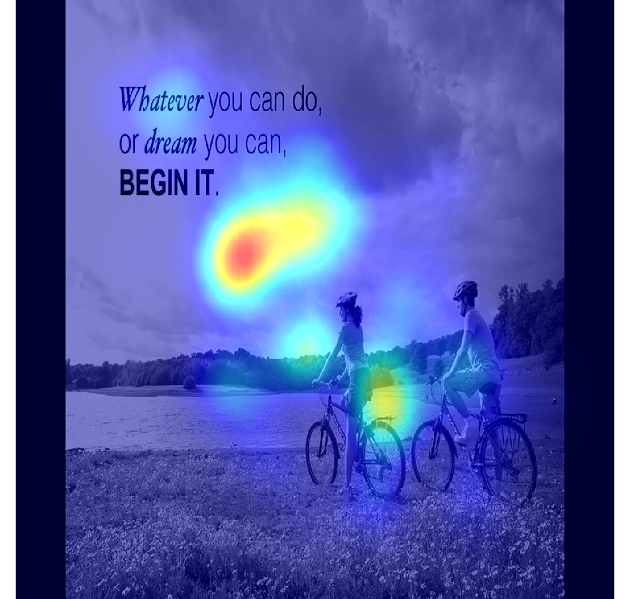 | 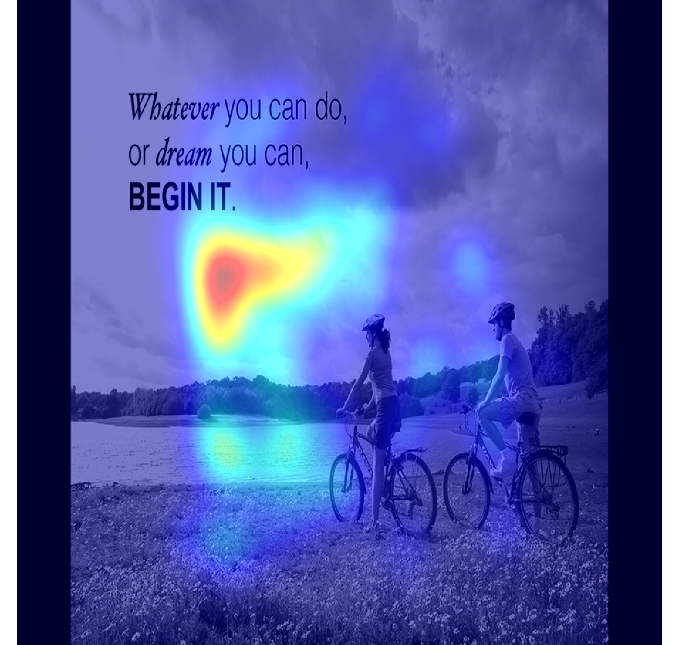 |

Supplementary material 4. Heat maps showing which regions of the materials had the most gaze points/longest gaze duration across participants with high versus low NFC.

Supplementary material 5: Linear regression models of the physical activity determinants outcomes with a pairwise interaction between need for cognition and intervention group.

|  |  | **Intentions** | | **Attitudes** | | **Perceived behavioral control** | |
| --- | --- | --- | --- | --- | --- | --- | --- |
|  |  | Coef [95% CI] | p-value | Coef [95% CI] | p-value | Coef [95% CI] | p-value |
| Intercept |  | 0.9 [-5.1, 7.0] | 0.76 | -2.2 [-7.6, 3.2] | 0.44 | 0.7 [-5.8, 7.2] | 0.83 |
| Baseline |  | 0.69 [0.53, 0.85] | <0.001 | 0.64 [0.51, 0.77] | <0.001 | 0.68 [0.47, 0.89] | <0.001 |
| Allocation  C v P | | -2.4 [-8.6, 3.8] | 0.45 | 1.8 [-3.5, 7.0] | 0.52 | -4.0 [-10.5, 2.5] | 0.23 |
| NFC |  | 0.02 [-0.05, 0.10] | 0.55 | 0.05 [-0.01, 0.12] | 0.12 | 0.00 [-0.08, 0.08] | 0.92 |
| Relevance |  | 0.24 [0.04, 0.44] | 0.02 | 0.41 [0.24, 0.58] | <0.001 | 0.32 [0.11, 0.54] | 0.01 |
| Age |  | -0.04 [-0.12, 0.04] | 0.37 | -0.05 [-0.12, 0.02] | 0.14 | -0.04 [-0.12, 0.05] | 0.40 |
| Gender  F v M | | -0.02 [-1.72, 1.69] | 0.98 | 0.31 [-1.20, 1.83] | 0.69 | 0.99 [-0.73, 2.71] | 0.27 |
| Prior Message Exposure |  | 0.33 [-0.50, 1.16] | 0.44 | 0.59 [-0.12, 1.29] | 0.11 | 0.10 [-0.78, 0.98] | 0.82 |
| MVPA^1^ |  | 0.0012 [-0.0013, 0.0036] | 0.36 | 0.0014 [-0.0007, 0.0035] | 0.20 | 0.0003 [-0.0023, 0.0030] | 0.80 |
| NFC x allocation |  | 0.03 [-0.07, 0.12] | 0.60 | -0.03 [-0.11, 0.05] | 0.48 | 0.06 [-0.05, 0.16] | 0.29 |

*^1^MVPA = moderate to vigorous physical activity reported at baseline, with vigorous activity minutes weighted by two.*
